# Preventing inflammation inhibits biopsy-mediated changes in tumor cell behavior

**DOI:** 10.1038/s41598-017-07660-4

**Published:** 2017-08-08

**Authors:** Maria Alieva, Andreia S. Margarido, Tamara Wieles, Erik R. Abels, Burcin Colak, Carla Boquetale, Herke Jan Noordmans, Tom J. Snijders, Marike L. Broekman, Jacco van Rheenen

**Affiliations:** 1 Cancer Genomics Netherlands, Hubrecht Institute-KNAW & University Medical Center Utrecht, Uppsalalaan 8, 3584CT Utrecht, The Netherlands; 2 Departments of Neurology, Massachusetts General Hospital, Harvard Medical School, 149 13th Street, Boston, MA 02129 USA; 3 Medical Technology and Clinical Physics, University Medical Center Utrecht, Heidelberglaan 100, 3584 CX Utrecht, The Netherlands; 4 Department of Neurology & Neurosurgery, Brain Center Rudolf Magnus, University Medical Center Utrecht, Heidelberglaan 100, 3584 CX Utrecht, The Netherlands

## Abstract

Although biopsies and tumor resection are prognostically beneficial for glioblastomas (GBM), potential negative effects have also been suggested. Here, using retrospective study of patients and intravital imaging of mice, we identify some of these negative aspects, including stimulation of proliferation and migration of non-resected tumor cells, and provide a strategy to prevent these adverse effects. By repeated high-resolution intravital microscopy, we show that biopsy-like injury in GBM induces migration and proliferation of tumor cells through chemokine (C-C motif) ligand 2 (CCL-2)-dependent recruitment of macrophages. Blocking macrophage recruitment or administrating dexamethasone, a commonly used glucocorticoid to prevent brain edema in GBM patients, suppressed the observed inflammatory response and subsequent tumor growth upon biopsy both in mice and in multifocal GBM patients. Taken together, our study suggests that inhibiting CCL-2-dependent recruitment of macrophages may further increase the clinical benefits from surgical and biopsy procedures.

## Introduction

In the clinic numerous biopsies are taken from cancer patients on a daily basis. Biopsies are indispensable for the correct diagnosis, prognosis and determination of optimal and personalized therapies based on the tumor’s (genetic) profile^1–3^. More invasive procedures, such as tumor resection, have been shown to potentiate the growth of the remaining tumor cells thereby potentially affecting tumor recurrence and metastasis formation^4–7^. Although not well-characterized, less invasive procedures, such as (needle) biopsies, may have similar effects^8^.

In non-pathological situations, wounded tissue is repaired by a cascade of cellular events, including the induction of an inflammatory response that promotes proliferation and migration of surrounding cells to close the wound^9, 10^. Yet, in cancer, proliferation and migration are two deleterious processes involved in tumor progression. In particular, for highly aggressive brain tumors such as glioblastoma multiforme (GBM), tumor growth and local dissemination lead to decreased survival times^11^. Since biopsies are the gold standard for diagnosis, inhibition of adverse side effects will further increase the clinical benefit of this procedure. To fully understand, whether and how biopsies affect the behavior of the remaining GBM cells, new techniques are required to allow the study of these potential effects in the physiological context of living organisms.

Here we show in a retrospective analysis of GBM patients that (needle) biopsy increases the tumor-volume. To identify the cellular mechanisms that mediate this response, we developed multi-day repeated high-resolution intravital microscopy (IVM) tools in mice and analyzed how tumor cell migration and proliferation rates change in response to biopsy over multiple days. Using our IVM tools, we show that biopsy in brain tumors of mice induces a CCL-2-dependent recruitment of macrophages that potentiates tumor cell migration and proliferation. In mice, we show that the biopsy-induced induction of tumor cell migration and proliferation is dependent on inflammation, especially on the recruitment of macrophages, and that it can be blocked by treatment with dexamethasone (DEX), a standard glucocorticoid given to GBM patients to prevent or treat brain edema. Indeed, our retrospective clinical analysis shows that DEX treatment prior to (needle) biopsy prevents the biopsy-induced tumor-volume increase in GBM patients.

## Results

### Clinical and experimental observation of biopsy-induced tumor progression

**Figure 1:**
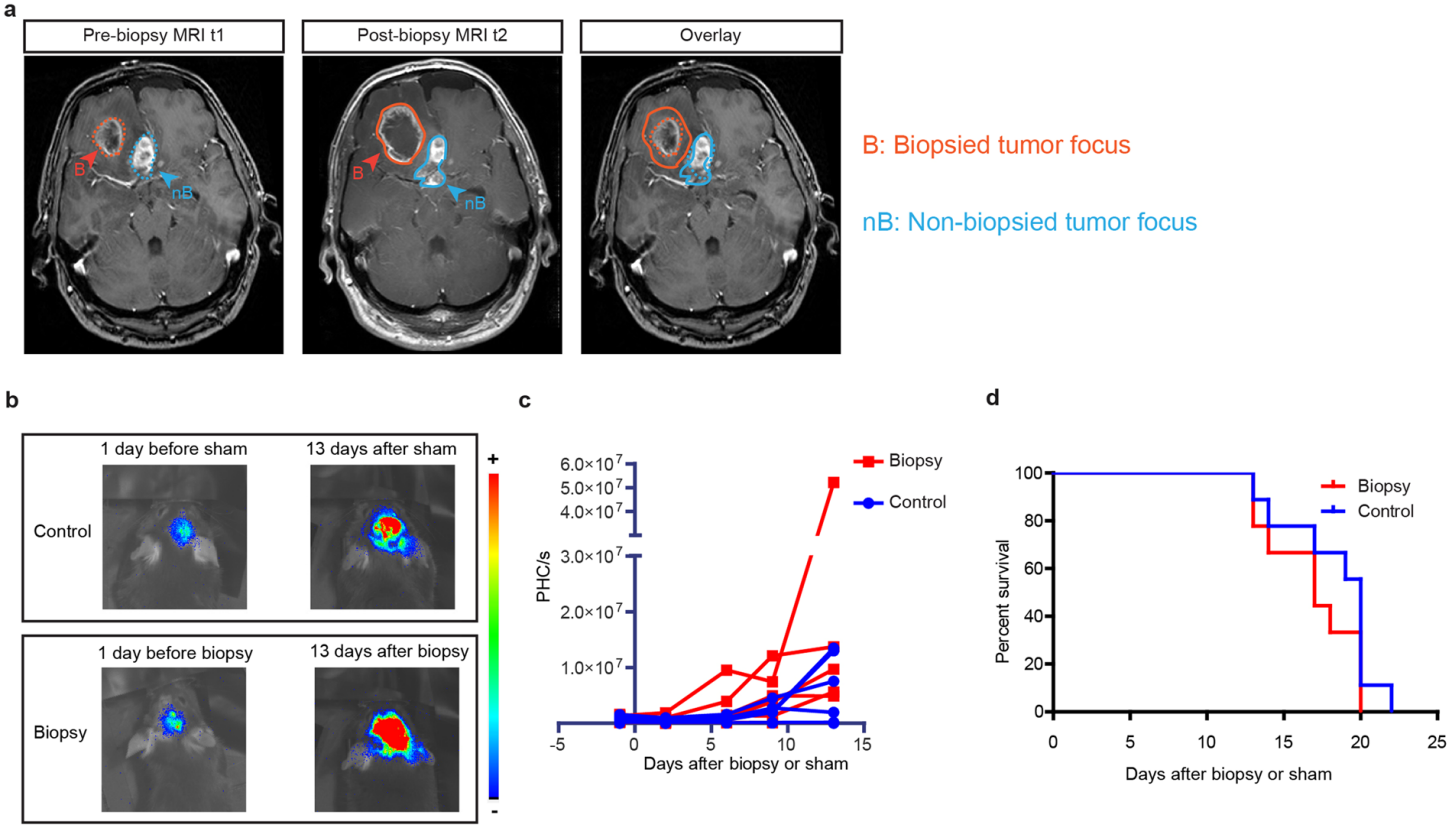
Effect of biopsy on glioma progression. (**a**) Representative slices of MR images taken before (left panel) and after biopsy (middle panel) showing the progression of the biopsied and non-biopsied tumors (right panel). (**b**) Representative images showing glioma progression in control and biopsied tumors monitored by bioluminescence. (**c**) Kinetics of glioma growth per individual mice. Each dot represents the photon counts per second (PHC/s) values from individual mice, values from the same mouse are connected with a line (n = 9). (**d**) Kaplan-Meier survival curves of mice that received a biopsy-like injury or control mice.

**Figure 2:**
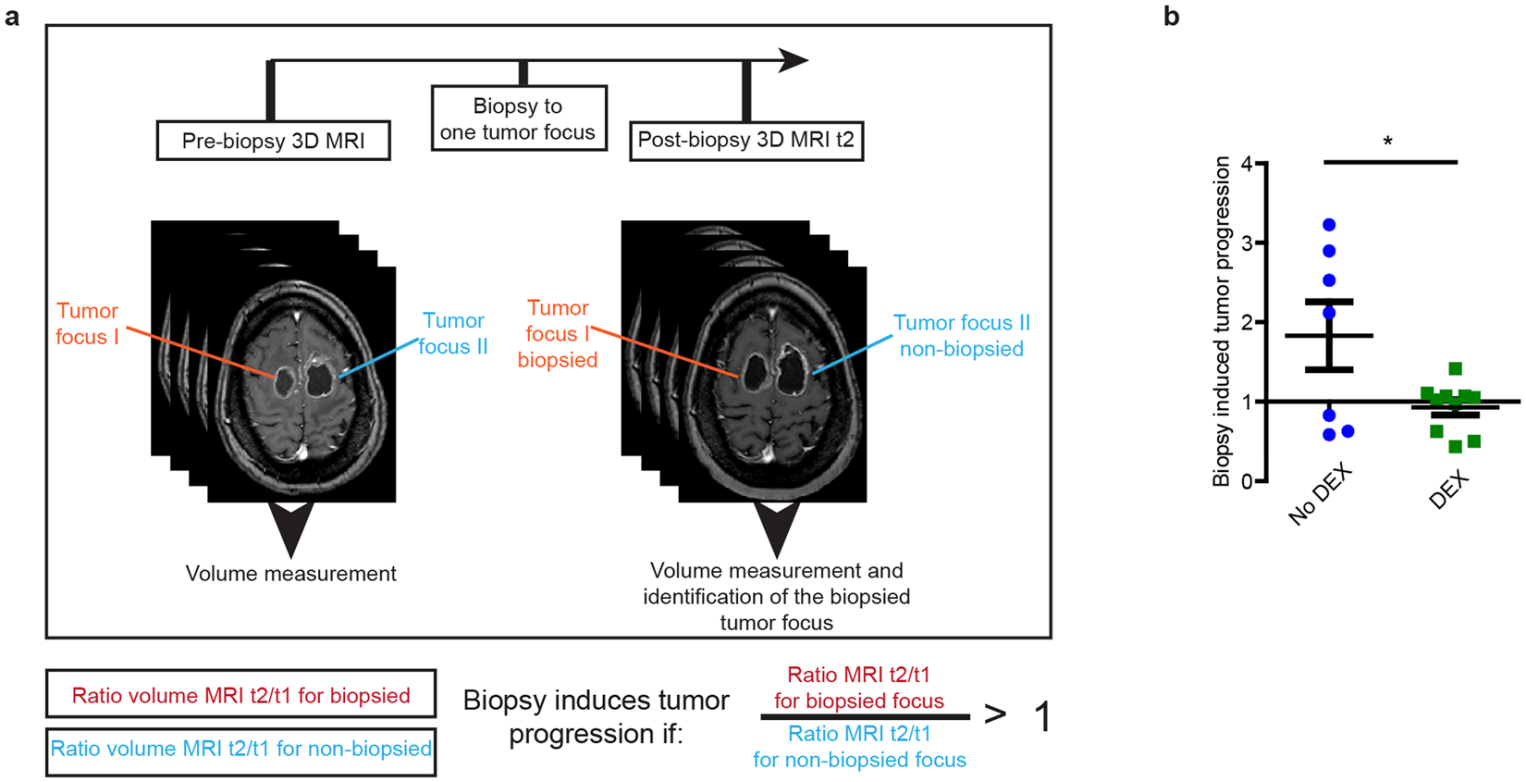
DEX treatment blocks biopsy-induced tumor progression in patients with multifocal GBM. (**a**) Cartoon showing MRI analysis setup. (**b**) The normalized tumor progression of biopsied tumor in patients that did not receive DEX before biopsy (blue circles) and patients that receive DEX before biopsy (green squares). Every symbol represents the mean of an individual patient. (*n* >= 7 per condition, *P < 0.05 versus control, Student’s *t* test).

To test whether a (needle) biopsy has an effect on tumor-volume in patients, we performed a retrospective analysis on a group of 785 GBM patients treated in our hospital over the last 10 years. We identified 21 patients with multifocal GBM (patients with several tumor foci in the brain), who underwent a biopsy in only one of the tumors and of which 3D magnetic resonance images (MRI) were taken before and after biopsy (Supplementary Table 1). We excluded 4 patients because either the T1-images before and after contrast injection, the T2/flair images, or diffusion-weighted images showed hemorrhage or ischemia. This analysis showed that in patients that did not receive anti-edema medication DEX prior to the biopsy, the volume of biopsied tumors increased more compared to non-biopsied tumors (Fig. 1a and Fig. 2b). To evaluate whether we could recapitulate this effect in mice, with more controlled experimental conditions, we monitored tumor growth of a murine GBM model. We orthotopically injected mouse glioma GL261 cells expressing a firefly luciferase in the brains of C57BL/6 mice. Upon injection, highly invasive tumors developed within a week. Mice were divided into groups with similar tumor sizes. In addition to survival time, tumor growth was monitored by bioluminescence. In two independent laboratories, we found that even in this very aggressive and fast growing tumor model, upon biopsy, tumors tended to grow faster (Fig. 1b,c; Supplementary Fig. 1) leading to slightly decreased survival times (Fig. 1d; Supplementary Fig. 1). This data suggests that this fast growing and aggressive murine GBM model can recapitulate the observations in patients.

### Intravital imaging of tumor cell migration and proliferation in glioblastoma multiforme

**Figure 3:**
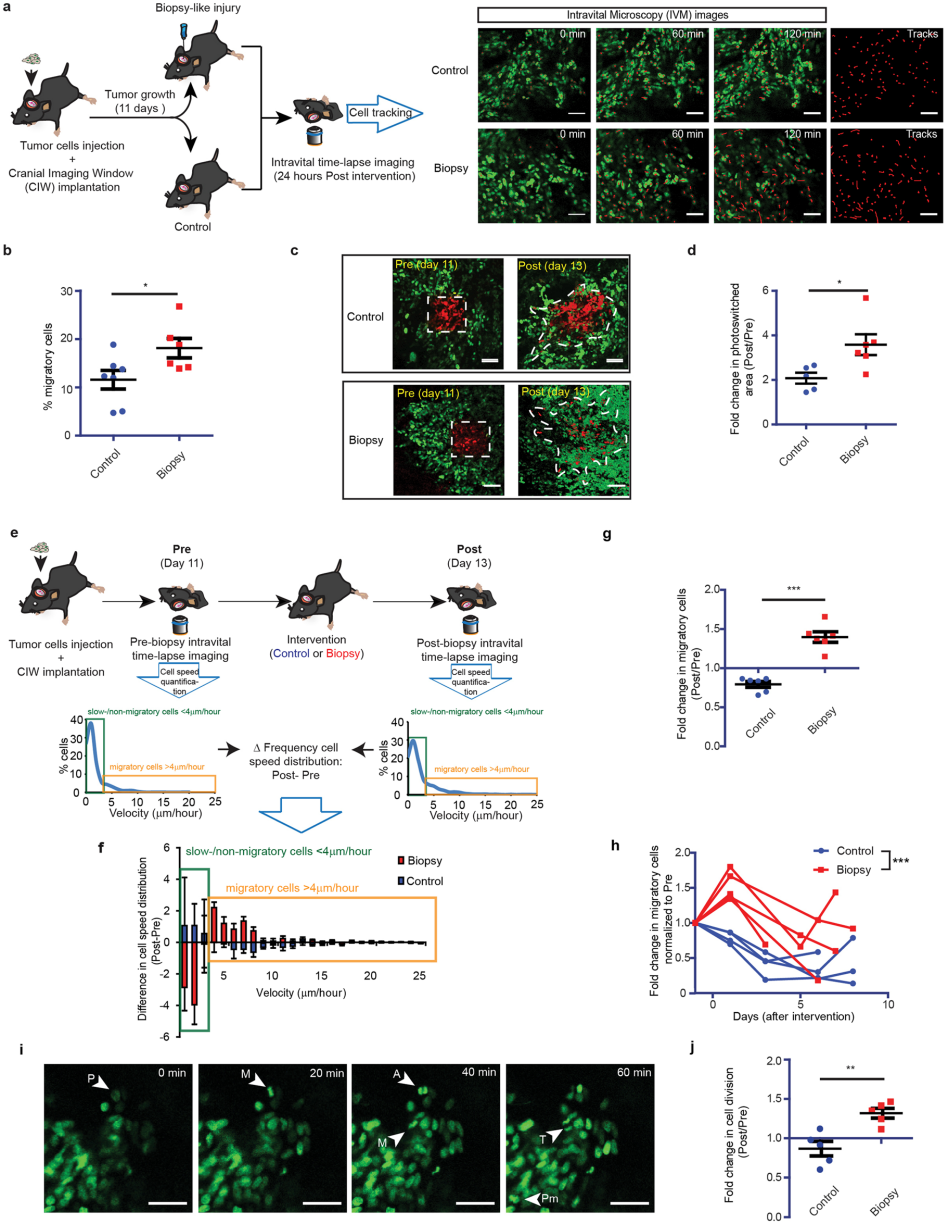
Change in tumor migratory and proliferative behavior upon biopsy revealed by repeated intravital imaging. (**a**) A schematic overview of the setup of the experiment. IVM images of biopsied and control GL261 H2B-Dendra2 tumors. Red lines highlight individual tumor cell tracks. Scale bar represents 50 µm. (**b**) Quantification of percentage of migratory tumor cells, where each dot represents the mean value of an individual mouse. (*n* >= 6 mice, *P < 0.05 versus control, Student’s *t* test) (**c**) Representative H﻿2B-Dendra2 images of tumor cell migration and infiltration. The white dotted line represents the migration and infiltration area. Scale bar represents 50 µm. (**d**) The increased photoconverted area plotted for the indicated conditions. Every dot represents the mean value of an individual mouse. (*n* >= 5 mice *P < 0.05 versus control, Student’s *t* test). (**e**) Cartoon showing experimental design. The waterfall plots are calculated by subtracting the cell velocity distribution pre-intervention from the cell velocity distribution post-intervention. (**f**) Waterfall plots showing the change in cell velocity distribution relative to basal migration in individual mice. The data is shown as mean ± S.E.M. of 5 mice. (**g**) The number of migratory cells in control (blue) and biopsy (red) animals normalized to the pre-intervention values. (*n* = 6 mice ***P < 0.0001 versus control, Student’s *t* test). (**h**) The normalized (relative to pre-intervention) number of migratory cells in individual mice over time. (*n* >= 4 mice per condition, ***P < 0.0001 versus control, two-way ANOVA). (**i**) Representative *in vivo* time-lapse images showing dividing cells in GL261 H2B-Dendra2 tumors. Different stages of mitosis are indicated: prophase (P), prometaphase (Pm), metaphase (M), anaphase (A), telophase (T). Scale bar represents 50 μm. (**j**) The normalized number of dividing cells in control (blue) and biopsy (red) animals. Per individual animal, the values Post intervention were normalized to the values Pre. (*n* = 5 mice, **P < 0.01 versus control, Student’s *t* test).

To reveal the mechanism of biopsy-induced tumor growth, we intravitally imaged the behavior of tumor cells before and after biopsy. We orthotopically injected GL261 cells expressing a nuclear fluorescent protein (H2B-Dendra2) in the brains of C57BL/6 mice and implanted a chronic cranial imaging window (CIW) above the injection site (Fig. 3a). Intravital images were acquired through the CIW at multiple time points using a multiphoton microscope to visualize migration of single tumor cells at high resolution up to a depth of several hundred micrometers. A series of time-lapse z-stack images of the tumor volume were acquired for 2 hours with a time interval of 20 minutes. In these movies, the movement of individual tumor cells was determined by tracking the migration path over time in different xy planes of the z-stack (Fig. 3a, Supplementary Fig. 2). As observed in other models^12^, most tumor cells showed no or slow migration, and only a minor fraction was motile (Fig. 3a,b; Supplementary Movie 1). Interestingly, 1.58 (SD = 0.51) times more motile tumor cells were found in tumors after biopsy-like injury compared to control tumors without intervention (Fig. 3a,b; Supplementary Movie 2).

Time-lapse imaging allows us to measure migration of individual cells for several hours. To quantify cell migratory capacity over a period of three days, we made use of the photo-convertibility of the fluorescent protein Dendra2 expressed in our tumor cells. Upon exposure to ultraviolet light Dendra2 irreversibly switches from green to red^13^. To monitor tumor cell migration and infiltration over multiple days, we photo-marked ~200 Dendra2-expressing tumor cells in a square region (Fig. 3c, red cells). Two days later, we retraced and analysed the photoconverted region, and found that photoconverted (red) tumor cells had migrated and infiltrated into the surrounding tumor tissue. In line with our time-lapse imaging experiments, we observed that the infiltration area was 1.72 (SD = 0.41) times larger in tumors after a biopsy-like injury compared to control tumors without intervention (Fig. 3c,d). Combined, these data show that biopsy-mediated wounding increases tumor cell migration and infiltration.

We observed a large variation in the number of migratory tumor cells between individual mice (Fig. 3b). To identify the effect of biopsy in individual animals, we compared the distribution of migration velocity before and after the intervention in the same tumor (Fig. 3e). The differences in distribution, as depicted in the waterfall plot, illustrate that upon biopsy the number of migratory cells (cells with velocity >4 µm/hr) increased with a concomitant decrease in the number of slow-/non-migratory cells (cells with velocity <4 µm/hr) (Fig. 3f; Supplementary Fig. 3). By contrast, in the non-intervention control group, we observed that over time the number of cells that migrate fast slightly decreased with a concomitant minor increase in the number of slow-/non-migratory cells (Fig. 3f; Supplementary Fig. 3a). On average in the same mouse, we observed a 1.75 (SD = 0.16) fold increase in percentage of migratory cells upon biopsy relative to the control (Fig. 3g). We also analyzed the directionality of the highly migratory and persistent tumor cells and found that overall tumor cell movement was not directed towards the biopsy-site (Supplementary Fig. 4c), suggesting that the observed effect was not due to wound closure, but potentiation of tumor cells motility. Successive intravital imaging sessions revealed that the percentage of migratory tumor cells eventually decreased over time in both control and biopsied mice, but up to at least one week after intervention tumor cells in biopsied mice still exhibited a higher migratory capacity than the control mice (Fig. 3h).

Next we tested whether biopsy-like injury also affects tumor cell proliferation. H2B-tagged Dendra2 enables us to identify mitotic cells in our intravital imaging movies, since chromosomes and therefore H2B-Dendra2 condense upon mitosis^14, 15^ (Fig. 3i). Strikingly, we found a 1.52 (SD = 0.26) fold increase in the number of mitotic events upon biopsy relative to the non-biopsied control (Fig. 3j; Supplementary Fig. 3b). By contrast, in the control group the percentage of dividing cells slightly decreased over time (Fig. 3j; Supplementary Fig. 3b). Since the induction of migration and proliferation of tumor cells was not observed in control mice that underwent replacement of the CIW without biopsy (Supplementary Fig. 4), our data indicates that in the remaining tumor cells proliferation and migration are promoted by the biopsy-like injury.

#### Biopsy-induced progression is localized to the site of injury

In a complementary approach we examined the effect of biopsy on breast tumor cell migration. Organoids generated from ductal mammary carcinomas from polyomavirus middle T antigen (PyMT) mice were transduced for stable expression of H2B-Dendra2 and injected in the mammary gland of non-obese diabetic SCID IL-2 receptor gamma chain knockout (NSG) mice. Upon tumor development, mice underwent a biopsy-like injury and tumors were imaged through a mammary imaging window (MIW). Due to the compact tumor structure of mammary tumors, we could identify the biopsy site in our movies and link the percentage of migrating cells to the distance to the biopsy site (Supplementary Fig. 5a). Notably, we found more migratory cells close to the biopsy (<1 mm) than at a distant sites (>1 mm) (Supplementary Fig. 5b). Additionally, Dendra2 photoconversion showed more tumor cell infiltration close to the biopsy (<1 mm) than at distant sites (>1 mm) (Supplementary Fig. 5c,d). This data suggests that biopsy-induced tumor cell migration is most pronounced close to the wounded site. Furthermore, our data indicates that the effect of biopsy on tumor cell behavior is not restricted to GBM tumors, but could be a general phenomenon.

### Biopsy-induced tumor cell progression is induced by CCL2-recruited macrophages

**Figure 4:**
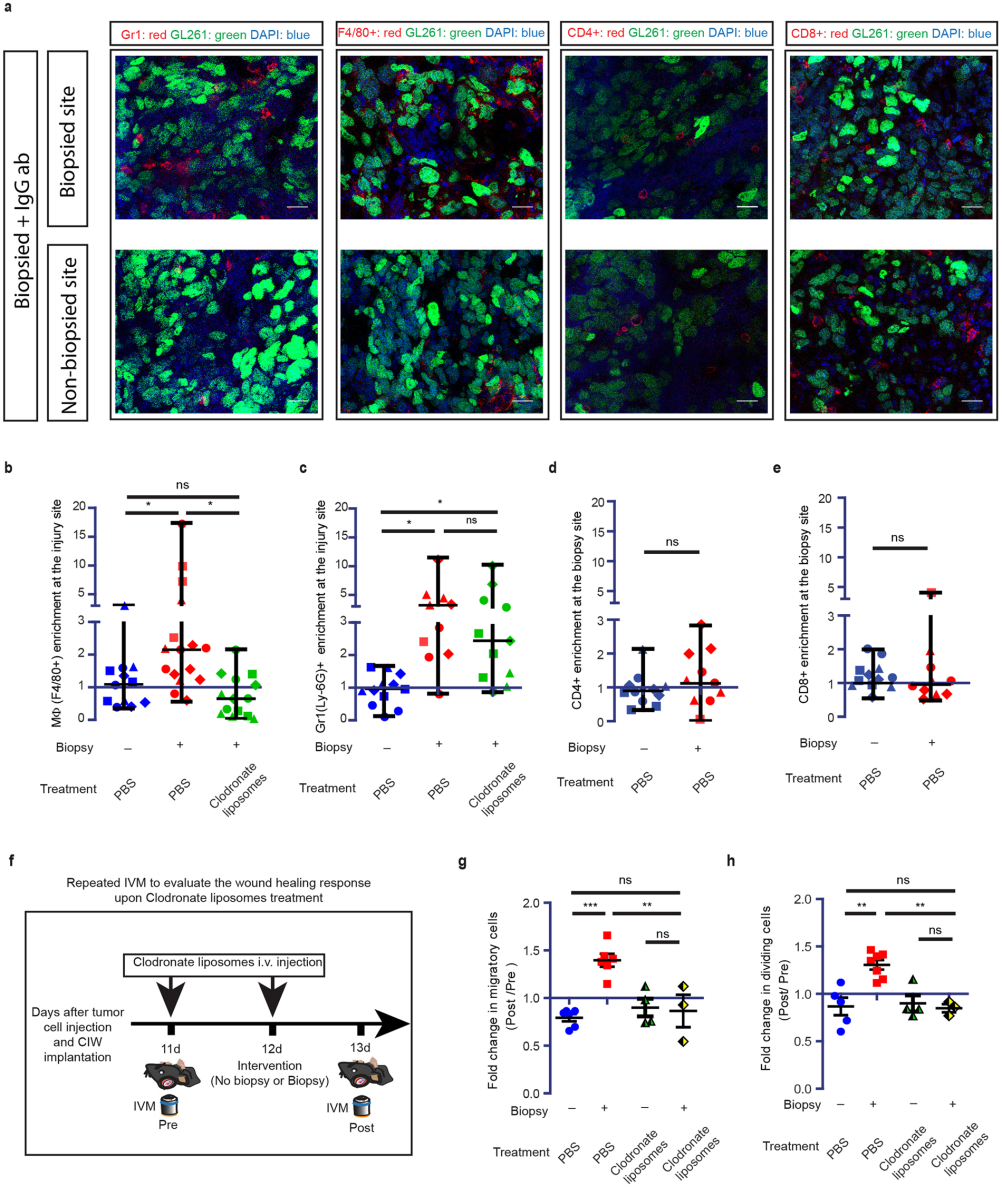
Monocyte recruitment is required for biopsy-induced tumor progression. (**a**) Representative staining showing Gr-1 (Ly-6G) + neutrophils; F4/80 + macrophages/microglia; CD4 + lymphocytes; CD8 + lymphocytes at biopsied and non-biopsied sites of mice that were injected with a random IgG antibody assessed by confocal microscopy. Shown are immune cell stainings in red, H2B-Dendra2 GL261 expression in green and DAPI staining in blue. Scale bar represents 20 μm. (**b–e**) Normalized number of F4/80 + (**b**) and Gr-1 (Ly-6G) + (**c**), cells in control, biopsied and biopsied + clodronate liposomes groups. Normalized number of CD4 + (**d**) and CD8 + (**e**), cells in control and biopsied groups. Per slide, the values of immune cells/area at the biopsied site were normalized to the values outside the biopsied site. Symbols represent different mice. P value was calculated using non-parametric t test. (*n* >= 3 per condition, **P < 0.01, one-way ANOVA with Newman-Keuls’s post hoc test). (**f**) Cartoon showing experimental design to evaluate the biopsy-induced response upon monocytes depletion. (**g,h**) The normalized number of migratory (**g**) and dividing (**h**) cells in control; biopsy and biopsy + clodronate liposomes groups. Per individual animal, the values *Post* intervention were normalized to the values *Pre*. (*n* >= 3 mice per condition, **P < 0.01, ***p < 0.001, one-way ANOVA with Newman-Keuls’s post hoc test).

To further increase the prognostic and diagnostic benefit of biopsies, we aimed to understand what mediates biopsy-induced tumor cell migration and proliferation, and focused on the first events that take place upon biopsy. Wounding leads to the acute induction of an inflammatory response consisting of immune cell activation and recruitment followed by pro-inflammatory chemokine secretion^9^. Therefore, we isolated GL261 tumors 24 hours after biopsy, and analyzed the recruitment of immune cells to the injury site by immunohistochemistry. In immunohistochemical sections the biopsy site was identified by the presence of red fluorescent nano-spheres that we injected during the biopsy. We quantified the number of immune cells in the biopsied area and compared it to the non-biopsied area on the same slide. Since immune cells are not homogenously distributed throughout tumors, we also analyzed the presence of immune cells in random areas of tumors in control non-biopsied mice. At the biopsy site we observed a 3.3 (SD = 0.88) fold increase in the number of Gr1^+^ neutrophils and a 2 (SD = 1.4) fold increase in the number of F4/80^+^ macrophages/microglia (Fig. 4a–c; Supplementary Fig. 6). As expected, later inflammatory mediators such as CD4^+^ and CD8^+^ lymphocytes were not recruited to the biopsy site at this early stage after biopsy (24 hours) (Fig. 4d,e).

Next, we wanted to identify the cell type that is responsible for the biopsy-induced progression of the tumor. Pro-inflammatory chemokines released by tumor-associated macrophages have been shown to drive malignant tumor cell behavior such as migration and proliferation^16–18^. To test whether the biopsy-mediated tumor progression was indeed mediated by the recruitment of macrophages, we aimed to specifically inhibit macrophage recruitment. In contrast to brain-residing microglia, newly recruited macrophages in GBM tumors are derived from blood-residing monocytes that infiltrate into the tissue^19, 20^. Intravenous injection of clodronate liposomes (CL) at two consecutive days resulted in successful depletion of circulating monocytes (Supplementary Fig. 7), as previously described^21^, but not the resident microglia/macrophages that were already present throughout the tumor (including non-biopsied areas)^22^. To block the recruitment of circulating monocytes/macrophages to the biopsy site, we intravenously injected CL 1 day before and immediately before biopsy (Fig. 4f). As expected, depletion of circulating monocytes prevented the accumulation of F4/80^+^ cells but not of Gr1^+^ neutrophils at the biopsy site (Fig. 4b,c, Supplementary Fig. 6). Importantly, by blocking the recruitment of new F4/80^+^ cells to the biopsy site, we no longer observed a biopsy-induced increase in the number of migratory or proliferative tumor cells (Fig. 4g,h). Together this data suggest that the recruitment of F4/80^+^ monocytes/macrophages mediates the biopsy-induced tumor cell migration and proliferation.

**Figure 5:**
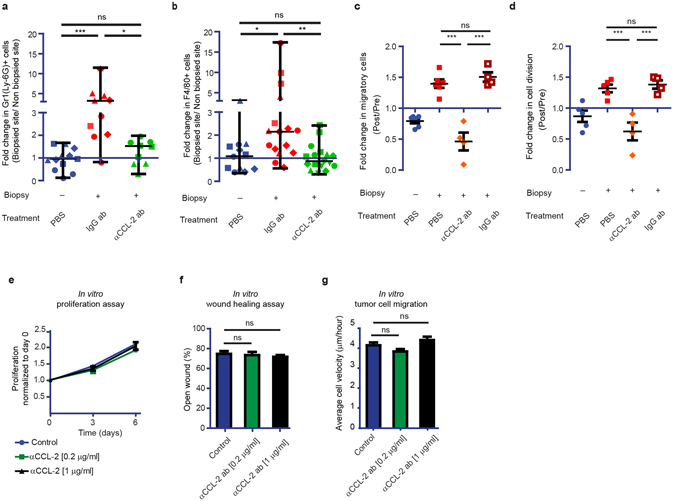
CCL-2 inhibition blocks biopsy-induced tumor progression. (**a,b**) Normalized number of Gr-1 (Ly-6G) + (**a**) and F4/80 + (**b**) cells in control; biopsy + IgG antibody; and biopsy + αCCL-2 antibody animals. Per slide, the values of immune cells/area at the biopsied site were normalized to the values outside the biopsied site. Symbols represent different mice. P value was calculated using non-parametric t test. *n* >= 3 per condition. (**c**,**d**) The normalized number of migratory (**c**) and dividing (**d**) cells in control; biopsy; biopsy + αCCL-2 antibody; and biopsy + IgG antibody animals. Per individual animal, the values *Post* intervention were normalized to the values *Pre*. *n* >= 4 mice per condition. ***P < 0.0001, one-way ANOVA with Newman-Keuls’s post hoc test. (**e**) *In vitro* cell proliferation of GL261 cells cultured with PBS or αCCL-2 antibody. Data is based on triplicates. (**f**) *In vitro* GL261 invasion measured by scratch assay of cells cultured with PBS or αCCL-2 antibody. Data is based on triplicates. (**g**) Graph showing the average velocity of individual GL261 cells tracked *in vitro*, cultured with PBS or αCCL-2 antibody. Data is based on triplicates.

Next, we tried to understand the molecular mechanism of the macrophages recruitment. In breast cancer the recruitment of inflammatory monocytes to primary and secondary tumor sites has been shown to be mediated by secretion of chemokine (C-C motif) ligand 2 (CCL-2) by tumor and stromal cells^23^. A recent report described that sterile hepatic injury triggers the early recruitment of pro-inflammatory monocytes expressing the receptor for CCL-2: CCR2^24^. In the central nervous system, this chemokine has been implicated in pathologies that display immune responses, such as traumatic brain injury^25^, ischemic stroke^26^ and multiple sclerosis^27^. To test whether CCL-2 mediates macrophage recruitment to the injury site, we injected an anti-CCL-2 neutralizing antibody locally at the biopsy site. Indeed, local blockade of CCL-2 prevented the recruitment of macrophages to the biopsy site (Fig. 5a,b; Supplementary Figs 6, 8). Next, we used IVM to test whether the blockage of the recruitment of macrophages changed biopsy-mediated tumor cell behavior. Similar to our finding upon depletion of macrophages, inhibition of macrophage recruitment to the biopsy-site using anti-CCL-2 neutralizing antibody resulted in a 3.25 (SD = 0.63) fold decrease in the number of migratory tumor cells compared to treatment with a control IgG antibody (Fig. 5c). In addition, blocking CCL-2 also led to a 2.3 (SD = 0.47) fold decrease in the number of proliferative cells (Fig. 5d). Importantly, *in vitro* tumor cell migration and proliferation were not inhibited by the CCL-2 neutralizing antibody excluding any direct effects of the anti-CCL-2 antibody on tumor cell behavior (Fig. 5e–g). Combined, our data suggest that biopsy-induced tumor progression is induced by a CCL-2 mediated recruitment of macrophages.

### Dexamethasone treatment inhibits biopsy-induced tumor cell progression

**Figure 6:**
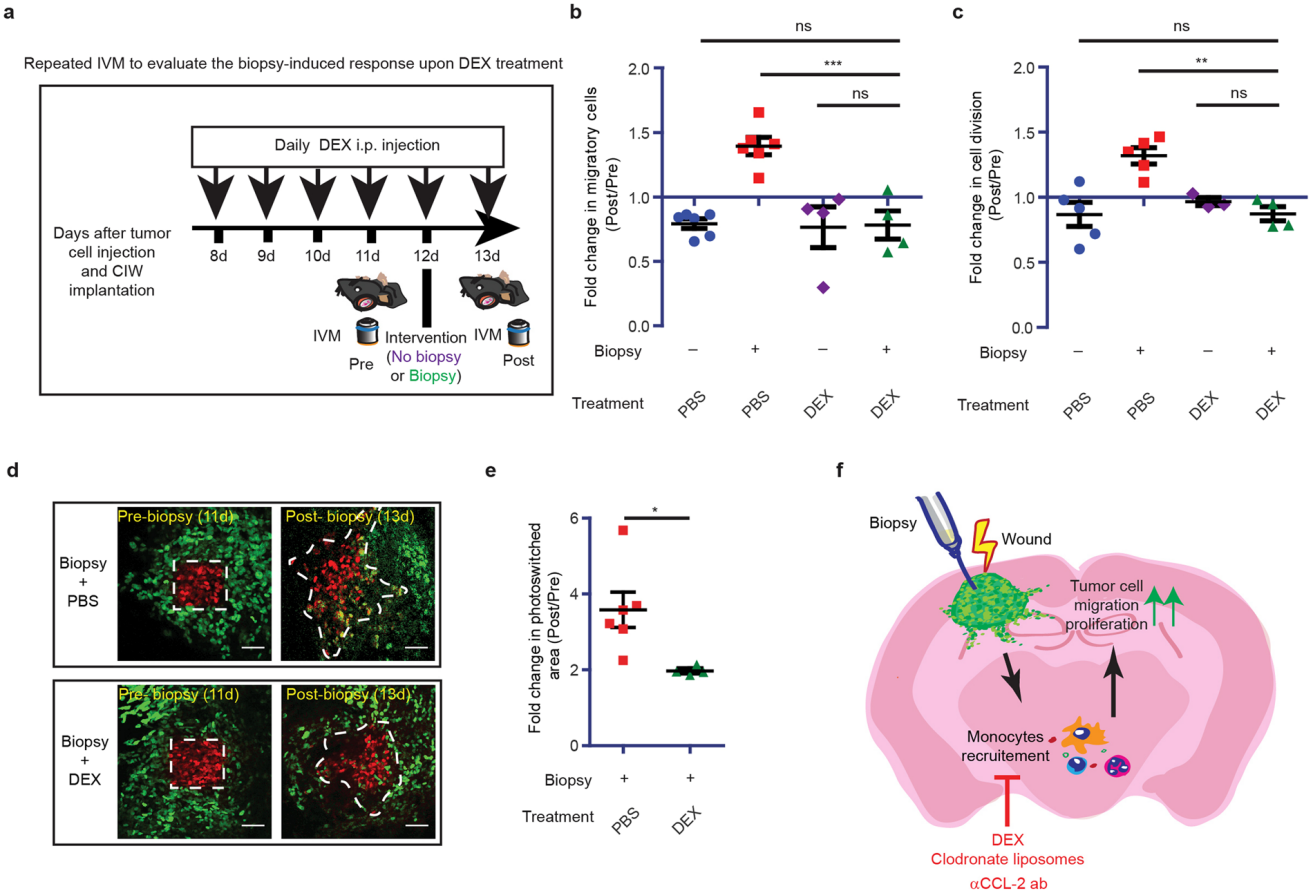
Biopsy-induced response is inhibited by DEX treatment. (**a**) Cartoon showing experimental design to evaluate the biopsy-induced response upon DEX treatment. (**b**) The increase in the number of migratory cells for the indicated conditions. Every symbol represents the mean of an individual mouse, and *n* >= 4 per condition. (**c**) The increase in the number of dividing cells for the indicated conditions. Every symbol represents the mean of an individual mouse, and *n* >= 3 per condition., **P < 0.01,***P < 0.001 one-way ANOVA with Newman-Keuls’s post hoc test. (**d**) The increased photoconverted area plotted for the indicated conditions. Every dot represents the mean value of an individual mouse. Scale bar represents 50 µm. (**e**) Quantification of increase in migration and infiltration area for the indicated conditions. The data is shown as mean ± S.E.M. *n* >= 4 mice, *P < 0.05 versus PBS group, Student’s *t* test. (**f**) Schematic illustration of a model showing how biopsy induces tumor cell migration and proliferation.

Glucocorticoids, such as DEX, are anti-inflammatory drugs commonly used in the clinic to prevent or treat brain edema^28^. To test whether DEX has the potential to inhibit the biopsy-induced response, we treated mice with DEX daily, starting 3 days before biopsy. In line with previous reports, DEX treatment reduced the numbers of circulating monocytes and other leukocytes in mice (Supplementary Fig. 8)^29^ and decreased the recruitment of F4/80^+^ macrophages/microglia and CD4^+^ and CD8^+^ lymphocytes to the tumors (Supplementary Fig. 9)^30, 31^. We found that suppressing inflammation by DEX treatment reduced the number of migratory and proliferative cells upon biopsy below control level (Supplementary Fig. 10). To exclude that increases in biopsy-induced migration and proliferation were masked by a general reduction in migration and proliferation due to DEX treatment, for every animal we normalized the levels of migration and proliferation after biopsy to the level before biopsy (Fig. 6a). We compared the results of biopsied animals treated with DEX (marked with green triangles) to the results from mice that received no biopsy and were treated with either DEX (purple diamonds) or PBS (blue circles), and did not observe significant differences in migration or proliferation between the three groups (Fig. 6b,c). Additionally, Dendra2 photoconversion showed that DEX treatment led to a 1.81 (SD = 0.32) fold decrease in tumor cell migration and infiltration upon biopsy compared to PBS treatment (Fig. 6d,e). Importantly, *in vitro* DEX treatment did not affect migration and proliferation of tumor cells (Supplementary Fig. 10c–e), suggesting that the *in vivo* action of DEX is mediated through inhibition of inflammation. Taken together, our results suggest that DEX treatment prior to biopsy inhibits the biopsy-mediated increase in the number of migratory and proliferative cells in addition to a general reduction in migration and proliferation (Fig. 6f).

### Dexamethasone treatment before biopsy blocks biopsy-induced tumor progression in multifocal GBM patients

To evaluate the impact of DEX treatment on biopsy-induced tumor progression in patients, we looked back at our retrospective analysis of a group of 785 GBM patients that have been treated in our hospital over the last 10 years. As mentioned above, 4 from the 21 patients with multifocal GBM were excluded since either the T1-images before and after contrast injection, the T2/flair images, or diffusion-weighted images showed hemorrhage or ischemia. We divided the 17 patients into two groups: 1) patients that received DEX treatment before the biopsy, and 2) patients that either did not receive DEX treatment or received DEX treatment after the biopsy. In line with our mouse data, we found using MRI that in patients that did not receive DEX before the intervention, the tumor volume increase was higher in the biopsied tumor compared to the non-biopsied tumor(s). Importantly, similar to our findings in mice, this effect was not observed in patients that had been pre-treated with DEX (Fig. 2a,b). To test for a relationship between faster progression and other co-variables in the two groups of patients, we compared other factors including initial tumor size and time between pre-biopsy and post-biopsy MRIs. Although we found a tendency that pre-treated patients have larger lesions and have a shorter time between the scans, these factors did not correlate with tumor progression (p = 0.34 and p = 0.80 respectively) (Supplementary Fig. 11). In the clinic biopsies are often taken from larger and potentially more “malignant appearing” tumors. Importantly, this selection process of biopsied lesions was the same for both the DEX pretreated group and the non-DEX pretreated group, and therefore could not have introduced a bias that would explain the observed differences between the two groups.

Combined our data suggest that in GBM patients, biopsy triggers the progression of tumors that can be prevented by pretreatment with a commonly used glucocorticoid, dexamethasone.

## Discussion

To date, biopsy followed by histology is still the gold standard and essential to obtain a definitive diagnosis of a tumor, and therefore the benefits of this procedure outweigh any potential unwanted side-effect. Nevertheless, the identification and subsequent inhibition of these potential side-effects may further increase the diagnostic and prognostic benefit of the procedure. Prior studies indicate that biopsy and surgical interventions can potentiate metastatic spreading and decrease patient survival^4, 8, 32–36^. However, the used approaches cannot identify the cellular mechanisms underlying biopsy-induced changes in tumor cell behavior. Large variations between individuals often disguise subtle but important changes in processes such as recruitment of immune cells and the induction of cancer cell migration and proliferation. Here, we have uncovered the effects of biopsy on the migration and proliferation of tumor cells by repeated imaging sessions in individual animals. We were able to validate our preclinical findings in a unique group of patients with multiple lesions (multifocal GBM). Within the same individual, we showed that, biopsied tumors tended to progress more, relative to non-biopsied lesions, and in addition that this effect was inhibited by dexamethasone treatment prior to biopsy. Moreover, these data combined with bioluminescent monitoring of tumor progression in living mice provide evidence that biopsy-induced changes in tumor cell behavior, shortly after biopsy, result in increased tumor progression at mid/long-term.

Our study shows that biopsy procedure has the potential to induce local recruitment of monocytes/macrophages, which subsequently induces tumor progression. In the last decade, it was well described that macrophages secrete matrix-remodeling proteins, cytokines and chemokines (e.g. MMP, TNF-α, EGF, TGFβ) that can induce the migration and proliferation of tumor cells^16–18, 37–39^. However, we cannot exclude that macrophages use additional mechanisms to alter tumor cell migration or spreading^40, 41^. In addition to these micro-environmental factors, the behavior of tumor cells is also strongly influenced by the genetic profile of cells (intrinsic capacities). Therefore our observed effects of biopsy may only hold true for late stage tumors, such as our mouse glioma and carcinoma models, in which cells have the intrinsic ability to migrate^42^. In contrast, in more benign tumors, a biopsy may induce changes in the microenvironment; however, migration may not be triggered because cells lack the intrinsic capacity that enables them to migrate. In line with this idea, the migratory capacity of non-tumoral astrocytes was previously found not to be affected by brain injury^43^. However, a recent study from Wijnenga and colleagues indicate that in low-grade glioma patients, biopsy can also decrease survival^35^, which opposes this idea.

Our findings show that pretreating patients with dexamethasone, a commonly used glucocorticoid, can prevent the local, biopsy-induced inflammation and progression of tumors. In line with previous reports we found that dexamethasone treatment decreased the levels of circulating monocytes as well as other immune cells in mice^29^. Although in humans dexamethasone treatment has been reported to increase neutrophil numbers, it is important to note that this is mainly due to demargination of intravascular neutrophils and not actual increase in the numbers of circulating cells^44^. Moreover, dexamethasone not only changes the number of immune cells but also has an inhibitory impact on a wide range of immune responses^45^ and it has been suggested that it could have adverse effects if combined with radio or chemotherapy^46, 47^. To fine-tune the dexamethasone treatment in a biopsy situation, additional *in vivo* studies are needed to investigate the exact nature of the biopsy-induced macrophages, their recruitment mechanisms and the impact of dexamethasone on macrophage subtype/polarization. For example, dexamethasone can potentially also inhibit classically activated macrophages that have been described to have anti-tumoral effects^48^.

It is important to point out that, although some clinicians routinely give dexamethasone to patients prior to a biopsy, protocols vary per hospital. Furthermore, in some cases patients cannot receive dexamethasone, for instance due to the history of psychiatric illness or unwanted effects on the histological accuracy that could lead to incorrect diagnosis (e.g. suspected lymphoma). Our experiments with inhibition of the CCL-2 chemokine showed that the local injection of anti-inflammatory antibodies at the biopsy site immediately after the procedure is an alternative strategy to inhibit biopsy-induced tumor cell migration and proliferation. These results open a possibility for the development of alternative therapeutic approaches based on the local inhibition following biopsy to allow an accurate diagnosis of the lesion. Moreover, it should be considered to take advantage of biopsy-induced inflammation for the treatment of cancer, by combining biopsy with a therapy that can alleviate immune suppression at the tumor microenvironment while boosting both innate and adaptive tumor-reactive immune responses^49^.

Our study is mainly based on murine models and a retrospective study on a small cohort of patients, and therefore it is too early to draw conclusions how to optimize the biopsy procedure in the clinic. For this, future (multicenter) studies with a larger cohort of multifocal GBM patients and standardized 3D MRI are required. The need of such an effort is further demonstrated by a recent study showing an association of biopsy with shorted survival in low-grade glioma patients^35^. Moreover, given the extremely poor prognosis that GBM patients face, every improvement in survival can be viewed as a big step forward for these patients.

## Materials and Methods

### Cell culture and stable cell line generation

GL261 cells were cultured in Dulbecco’s Modified Eagle’s Medium + GlutaMAX (DMEM; GIBCO, Invitrogen Life Technologies, Paisley, UK) supplemented with 10% (v/v) fetal bovine serum (Sigma, St. Louis, MO, USA), 100 μg/ml streptomycin, and 100 U/ml penicillin (Invitrogen Life Technologies, Paisley, UK). Cells were kept at 37 °C in a humidified atmosphere containing 5% CO_2_. The GL261 H2B-Dendra2 cell line was generated using a standard lentiviral transduction protocol with a pLV CMV-H2B-Dendra2 vector. Afterwards, transduced cells were selected using puromycin (Gibco Life Technologies, Paisley, UK). GL261 Fluc-GFP cell line was generated using a standard lentiviral transduction protocol with CSCW2 lentivirus vector carrying an expression cassette for firefly luciferase (Fluc) and GFP fluorescent protein separated by an internal ribosome entry site domain. Selection of transduced cells was done based on GFP expression using FACS (BD FACSAria II SORP Cell Sorter). Cells were expanded over one passage and injected in mice.

### Mice

C57BL/6 and NSG mice between 8–12 weeks old were used for experiments. C57BL/6 mice were housed under standard laboratory conditions and NSG mice (own colony) were housed under IVC conditions. Mice received food and water ad libitum. All experiments were carried out in accordance with the guidelines of the Animal Welfare Committee of the Royal Netherlands Academy of Arts and Sciences, the Netherlands and Massachusetts General Hospital, United States. The experimental protocols used in this manuscript were approved by a the Centrale Commissie Dierproeven (CCD) and the Instantie voor Dierenwelzijn (IvD).

### Tumor cell implantations for bioluminescent monitoring

For bioluminescent monitoring of tumor growth GL261 cells stably expressing a lentiviral vector for Fluc and GFP were injected in the brain of C57BL/6 mice. Mice were sedated with Hypnorm (Fluanison [neuroleptic] + Fentanyl [opioid]) (0.4 ml/kg) + Midazolam [benzodiazepine sedative] (2 mg/kg) at a dose of 1:1:2 in sterile water. Mice were mounted in a stereotactic frame and the head was secured using a nose clamp and two ear bars. Next, the top part of the head was shaved and an incision was made in the skin. A circular hole of 5mm diameter was drilled over the right parietal bone and 1 × 10^5^ GL261 H2B-Dendra2 cells suspended in 3ul of PBS were injected in the middle of the craniotomy at a 250 nl/min rate using a 10 μl Hamilton syringe with a 2 pt style needle at a depth of 0.5mm. The scalp was closed by Hystoacryl glue (Braun) and a single dose of 100 μg/kg of buprenorphine (Temgesic, BD pharmaceutical limited) was administered. After surgery the mice were provided food and water *ad libitum*. Furthermore, mice were closely monitored twice per week for behavior, reactivity and appearance.

### Bioluminescent monitoring

Tumor progression upon biopsy was monitored by bioluminescence as described previously^50^. Mice were sedated using isoflurane inhalation anesthesia (1.5% to 2% isoflurane/O_2_ mixture) and injected intra-peritoneal (i.p). with 150 μl of D-luciferin (Thermofisher) (15 mg/ml in saline). Animals were placed in a darkbox chamber of the Aequoria luminescence imaging system (Hamamatsu Photonics) and images were acquired 15 min after injection. A second image of the animal was obtained using a white-light source inside the detection chamber, to register the position of the luminescence signal. Mice were imaged twice per week after biopsy or sham was taken. Quantification and analysis of photons recorded in images was done using the Wasabi image analysis software (Hamamatsu Photonics).

### Survival analysis

To test the effect of biopsy on survival, mice were monitored on a daily based starting from day 10 after biopsy. Animals losing >20% of their initial body weight, losing >15% in two days, hunched posture or rough hair coat were sacrificed.

### Tumor cell injection and CIW surgery

Tumor cell injection and CIW surgery were performed at the same day as described previously^51^. In short, mice were sedated with Hypnorm (Fluanison [neuroleptic] + Fentanyl [opioid]) (0.4 ml/kg) + Midazolam [benzodiazepine sedative] (2 mg/kg) at a dose of 1:1:2 in sterile water. Mice were mounted in a stereotactic frame and the head was secured using a nose clamp and two ear bars. Next, the top part of the head was shaved and the skin was cut in a circular manner. After scraping the periosteum underneath and applying a drop of Xylocaine (Lidocaine 1% + Epinephrine 1:100,000) a circular groove of 5mm diameter was drilled over the right parietal bone. The bone flap was lifted under a drop of cortex buffer (125 mM NaCl, 5 mM KCl, 10 mM glucose, 10 mM HEPES buffer, 2 mM MgSO4 and 2 mM CaCl2, pH 7.4). Using thin forceps the dura mater was removed. Gelfoam sponge (Pfizer) was used to stop bleeding. Next, 1 × 10^5^ GL261 H2B-Dendra2 cells suspended in 3 μl of PBS were injected stereotactically using a 10 μl Hamilton syringe with a 2 pt style needle in the middle of the craniotomy at a depth of 0.5 mm. The exposed brain was sealed with silicone oil and a 6-mm coverslip glued on top. Dental acrylic cement (Vertex) was applied on the skull surface, covering the edge of the coverslip and a stainless steel ring was glued around the coverslip, to provide fixation to the microscope. A single dose of 100 μg/kg of buprenorphine (Temgesic, BD pharmaceutical limited) was administered. After surgery the mice were provided food and water *ad libitum*. Furthermore, mice were closely monitored twice per week for behavior, reactivity and appearance.

### Biopsy-like injury

At day 12 after tumor implantation and CIW mice from the biopsy and CIW replacement group received an intervention. Mice were sedated using isoflurane inhalation anesthesia (1.5% to 2% isoflurane/O_2_ mixture) and mounted in a stereotactic frame. The window was broken and the exposed cortical surface was kept moist with cortex buffer. In the biopsy group four injuries to the tumor area were made by means of a 25 G needle puncture at a depth of 1mm (Fig. 1a). In PyMT tumors one biopsy was performed using a 27 G needle puncture. In the CIW replacement group the window was broken and replaced but no biopsy was performed. In all groups the exposed brain was sealed with silicone oil and a 6-mm coverslip glued on top. After surgery the mice were provided food and water *ad libitum*.

Upon bioluminescent monitoring of tumor-progression the biopsy-like injury was performed similarly. Mice were sedated using isoflurane inhalation anesthesia (1.5% to 2% isoflurane/O_2_ mixture) and mounted in a stereotactic frame. An incision was made in the skin above the injection region. Tumor area was identified by GFP expression under a fluorescent stereotactic microscope (Leica) and in the biopsy group a biopsy-like injury was performed as described above. In the control group a sham incision was made but no biopsy-like injury was performed. The skin was sealed with hystoacril glue and the mice recovered on a heating pad.

### Intravital imaging

Mice were sedated using isoflurane inhalation anesthesia and placed face-up in a custom-designed imaging box. Small magnets embed in the imaging box facilitated CIW fixation. The isoflurane was introduced through the facemask, and ventilated by an outlet on the other side of the box. Time-lapse images of several positions of the tumor volume were acquired every 20 minutes for a maximum of 2 hours for GBM tumors and every 30 minutes for a maximum of 5 hours for PyMT tumors, during which the climate chamber surrounding the microscope was kept at 37 °C. For each position, images of the complete z stack of the tumor were acquired to a depth of 300 μm,with a step size of 3 µm (typically 70–100 images were acquired in each z stack). Imaging was performed on an inverted Leica TCS SP5 AOBS two-photon microscope (Mannheim, Germany) with a chameleon Ti:Sapphire pumped optical parametric oscillator (Coherent Inc.) and an inverted Leica SP8 multiphoton microscope with a chameleon Vision-S (Coherent Inc., Santa Clare, CA, www.coherent.com). Both microscopes are equipped with a 25x (HCX IRAPO NA0.95 WD 2.5mm) water objective with four non-descanned detectors (NDDs). The NDDs detected the following wavelengths: NDD1 <455 nm, NDD2 455–505 nm, NDD3 500–550 nm, NDD4 555–680 nm. Green H2B-Dendra2 was excited with 960 nm and detected with NDD3. Red H2B-Dendra2 was excited with 1020 nm and detected with NDD4. Sequential scanning was performed to detect green and red H2B-Dendra2. Scanning was performed in a bidirectional mode at 400 Hz and 12 bit, with a zoom of 1.3 for GBM and zoom of 1 for PyMT model, 512 × 512 pixels.

### Tracking migration of tumor cells

After imaging, acquired z-stacks were corrected for *z* and *xy* shifts using a custom-designed Visual Basic software program and further processed with Match motion compensation software program to correct for rigid and elastic tissue deformation^52^. Throughout the complete z stack we made maximum projections of 3 consequent z planes for the analysis, thus covering the complete imaging depth (Fig. S3). Up to 50/70 cells (for GBM and PyMT respectively) per imaging field of each z-projection and 1000 to 3000 cells per mouse and day were tracked manually with an ImageJ plugin (“MTrackJ” Rasband, W.S., ImageJ, U. S. NIH, Bethesda, Maryland, USA). The distance between z planes was of 3 μm, thus each maximum projection represented a volume of 6 μm, more than the size of one cell nucleus (a nuclear Dendra2 was used to mark the cells). This allowed us to track the cells that moved in the x and y planes in a volume of 6 μm. The analysis was performed in z projections obtain from all depths of the z-stack. Thus, although we tracked the cells in x and y planes, we obtained data on the migratory capacity of the cells from all the z-stack. At the beginning of each movie, a random cell was selected. The XY position was determined over time and the displacement for each individual cell was calculated by Excel (Microsoft).

### Analysis of tumor cell direction

At positions were the biopsy site could be identified highly migratory (average cells speed 8,8 μm/hour; average persistence 0,94) cells were tracked, as described above. The angle of tumor cell movement relative to the biopsy site was determined and the percentage of tumor cells migrating in a corresponding angular bin was quantified and represented in a wind-rose plot.

### Dendra 2 photoswitching

Dendra2 photoswitching was performed as previously described^53, 54^. Briefly, ROI scan option of LAS AF Lite software was used to photoswitch a chosen population of cells using the 405 nm laser line (20% power, 20–40 scans). Red form of the protein was collected to monitor the switching. Custom-designed Visual Basic and ImageJ software were used to enhance the red signal from the photoswitched cells.

### Analysis of cell division in IVM images

The number of dividing cells was analyzed as described before^14, 15^. Briefly, the number of mitotic figures were identified in a single IVM image acquired in a fixed time span and normalized to the total number of cells in this image.

### *In vivo* DEX and clodronate liposomes treatment

To test the effect of DEX on tumor cell behavior, mice received daily i.p. injections of DEX at 0.7 mg per kg body weight from day 8 after tumor cell implantation to day 13. Control mice received PBS. To deplete circulating monocytes, mice received two intra-venous injections of 100 µl of clodronate liposomes at 5 mg/ml (Clodronate liposomes.com). The injections were performed one day before biopsy and immediately before biopsy.

### Immunostaining

Gr-1 (Ly-6G) (RB6–8C5 clone, specific for neutrophils), CD4, CD8 and F4/80 stainings on 14 µm brain tissue cryosections were performed using corresponding mouse antibodies (rat anti-mouse Ly-6G clone RB6-8C5, BD Biosciences, rat anti-mouse CD4 eFluor660 clone GK1.5; rat anti-mouse CD8 eFluor660 clone 53-6.7; rat anti-mouse F4/80 biotin-conjugated clone mf48015, Invitrogen) overnight, followed by goat anti-rat antibody labelled antibody (A21247, Life Technologies) or labelled streptavidin (S-21374, Life Technologies).

### Immune cell quantification

14 μm thick cryosections were immunostained as described in section “Immunohistochemisty”. Tile scans of the tumor were acquired using a Leica DM6000 fluorescence microscope. Immune cells present in the tumor mass were quantified in tile scans of tumors using ImageJ plugins “Cell counter” and “Tumor margin”. To trace the biopsy site red fluorescent 1 μm beads (Thermofisher) were injected together with the IgG antibody. Tile scans of the tumor were acquired using a Leica SPE confocal microscope. Immune cells present at the biopsied and non-biopsied site were quantified using LasAF software.

### *In vitro* cell proliferation assay

Triplicates of 5 × 10^3^ GL261 cells were plated into 96-well plates and incubated overnight at 37 °C. Culture medium was replaced with medium containing 0, 1,10 µg/ml of dexamethasone. Cell proliferation was evaluated spectrophotometrically at 490 nm, using the CellTiter 96 Aqueous One Solution Cell Proliferation Assay (Promega) at days 1, 4 and 7. Results were expressed as the percentage of proliferation, where the proliferation of cells in culture medium at day 1 was set to 100%. Medium was refreshed every 3 days.

### Scratch assay

Triplicates of 10^5^ GL261 cells were plated into 96-well plates and incubated overnight at 37 °C. Culture medium was replaced with medium containing 0, 1, 10 µg/ml of Dexamethasone. When cells reached confluent monolayers, cultures were treated with 0.1 mg/ml of mitomycin C and wounded with the tip of a micropipette. Wound closure was imaged during 3 days using an inverted Leica AF7000 microscope equipped with a digital camera (Mannheim, Germany). The individual gaps were measured in each culture condition and at different time points. The residual gap between the migrating cells from the opposing wound edge was expressed as a percentage of the initial scraped area.

### *In vitro* migration assay

Cells were plated in triplicates in 48-well plates and incubated for 3 days with the conditions indicated above (Scratch assay). When cells reached 25% confluence, cultures were treated with 0.1 mg/ml of mytomicin C and imaged for 36 hours using an inverted Leica AF7000 microscope equipped with a digital camera. At least 200 cells per condition were tracked using ImageJ plugin (“MTrackJ”). Individual cell velocity was determined and the mean value and S.E.M. for pooled cells from the different replicates were calculated.

### Multifocal GBM patients

A total of 785 patients with a diagnosis of GBM between 2005 and 2015 were identified. Of these patients, 21 had multifocal GBM and had undergone a 3D T1 gadolinium MRI scan before and after biopsy and were included as the study sample for this article. Multifocal disease was defined as multiple tumor sites with clear separation between foci. The surgical intent was to biopsy one tumor focus, in accordance with the policy at our institution. The tumor volume of biopsied and non-biopsied tumor foci was recorded from the MRI pre-biopsy and in the follow up MRI after biopsy using OsiriX Lite. Tumor progression of a biopsied and a non-biopsied tumor in the same patient was measured by calculating the increase in volume from the pre-biopsy MRI to the post-biopsy MRI. The analysis was performed in a blinded manner by researchers that were blinded for the treatment that the patient received or if a tumor was biopsied or not. The use of patient data for this study was submitted to the ethics committee of our institution (Medisch Ethische Toetsingscommissie) and was considered exempt from full review.

Since increases of the size of biopsied tumors on T1-CE images may also be due to biopsy-related effects e.g. vascular damage causing hemorrhage or ischemia, we reviewed all relevant post-biopsy MRI images, including T1-images before and after contrast injection, T2/FLAIR images and diffusion-weighted images (DWI). We compared the post-biopsy images with the pre-operative images to detect signs of new hemorrhage (on pre-contrast T1 or DWI) or ischemia (on DWI and FLAIR) and excluded 4 patients that presented with hemorrhage after biopsy.

### Statistics

Results are shown as means as ± S.E.M. For all normal distributed measurements the Student’s t test (when two means were compared) or one-way ANOVA (when > 2 means were compared) test were used to determine if there was a significant difference between two means (P < 0.05), and for all others the Mann-Whitney U-test was used. A two-way ANOVA was also conducted to evaluate the long term effect of biopsy on tumor cell migration. Association of the biopsied tumor relative progression and the time between MRIs was evaluated using Pearson correlation statistics. All P values were two-tailed. Significance is marked with one asterisk when the P value is equal to or smaller than 0.05, with two asterisks when the P value is equal to or smaller than 0.01 and with three asterisks when the P value is equal or smaller the 0.001. Error bars are presented as ± S.E.M. All statistical analysis was performed using GraphPad.

### Data availability

The authors declare that all data supporting the findings of this study are available within the article and its Supplementary Information files, or from the corresponding author upon request.

## Electronic supplementary material

Supplementary Information
