# Supplementary material for: Preventing inflammation inhibits biopsy-mediated changes in tumor cell behavior
Source: Sci Rep. 2017 Aug 8;7:7529. doi: 10.1038/s41598-017-07660-4 (PMC5548904; doi:10.1038/s41598-017-07660-4)
Supplement: Supplementary file 1 — Supplementary Information [file 41598_2017_7660_MOESM1_ESM.doc]

**SUPPLEMENTARY MATERIAL AND METHODS**

**Title:** Preventing inflammation inhibits biopsy-mediated changes in tumor cell behavior.

**Authors:** Maria Alieva, Andreia S. Margarido, Tamara Wieles, Erik R. Abels, Burcin Colak, Carla Boquetale, Herke Jan Noordmans, Tom J. Snijders, Marike L. Broekman^2^, Jacco van Rheenen

**PyMT tumor organoids**

To establish PyMT tumor organoids, tumors were harvested and enzymatically digested using trypsin (from bovine pancreas, Sigma) and collagenase A (Roche) followed by several centrifugation steps until only the cell fragments of 200 to 1000 cells were left. These organoids were embedded in Basement Membrane Extract, Type 2 (RGF BME type 2 pathClear) and cultured in DMEM/F12 Glutamax supplemented with Hepes (1M Gibco), Penicillin-streptomycin, FGF (Life technologies) and B27 (50x Gibco). The PyMT H2B-Dendra2 organoids were generated by transduction with a pLV lentiviral vector for stable expression of CMV-H2B-Dendra2 using a lentiviral transduction protocol. Transduced organoids were selected using puromycin (Gibco Life Technologies, Paisley, UK).

**PyMT organoids orthotropic injection**

Organoids were collected and digested using trypsin up to 50-200 cells fragments. Organoids were suspended in Matrigel (Corning Matrigel Basement Membrane Matrix Growth Factor Reduced, Phenol Red Free) and injected into the fourth mammary gland of NSG mice. Tumors developed within 2-4 weeks and a mammary imaging window was surgically implanted one week before imaging.

**Mammary imaging window surgery**

The mammary imaging window was surgically implanted as described previously. In short, mice were anesthetized using 2% isoflurane/medical air anaesthesia. Surgical procedures were performed under aseptic conditions. Before surgery, the skin overlying the tumor was shaved and depilated, and the skin was disinfected using 70% EtOH. An incision was made through skin overlying the tumor and an imaging window was inserted. The imaging window was secured using a non-absorbable non-woven purse-string suture (4-0 prolene suture).

**Treatments of peripheral blood analysis.**

To test the effect of DEX on circulating immune cell counts, C57BL/6 mice received daily i.p. injections of DEX at 0.7mg per kg body weight for 5 days. Control mice received PBS. To test the effect clodronate liposomes on circulating immune cell counts, mice received two i.v. injections of 100µl of clodronate liposomes at 5mg/ml.

**Flow cytometry analysis on peripheral blood.**

Blood was collected via cardiac puncture and red blood cells were depleted using IOTest3 commercial lysis buffer (Beckman Coulter). Immune cells were spun down (4 minutes 500 RCF at RT) and counted to determine the total amount of immune cells blood. Blocking was performed for 10min in 80% FACS buffer (5 mM EDTA in PBS supplemented with 5% fetal calf serum) / 20% serum mix (50/50 normal goat serum (monx10961, Monosan) and FcγII/III receptor blocking serum 2.4G2 (Epirus Netherlands). Labelling was performed with the following antibodies: BV421 rat anti-mouse CD3 (clone 14A2 Biosciences), Alexa Fluor 488 rat anti-mouse CD45 (clone 30-F11 BioLegend), PE-Cy^TM^7 rat anti-mouse CD11b (clone M1/70 Biosciences), BV510 rat anti-mouse CD19 (clone 1D3 Biosciences), PE rat anti-mouse F4/80 (clone T45-2342 Biosciences), APC-Cy^TM^7 rat anti-mouse Ly-6G (clone 1A8 Biosciences) and BV711 rat anti-mouse Ly-6C (clone HK1.4 BioLegend). Samples were washed with FACS buffer and analysed on a FACS AriaII Special Ordered Reseach Product (BD Biosciences). The sort strategy is illustrated in Figure S9. Events acquired over stable flow were selected, followed by a gate excluding doublets. Immune cells were gated based on CD45 expression. A light scatter gate was constructed to exclude debris and non-viable cells from the analysis. The remaining events were represented in a F4/80 versus Ly-6G plot to identify neutrophils (F4/80neg, Ly6Gpos), F4/80pos, Ly6Gneg (monocytes/macrophages and eosinophils) and F4/80neg Ly6Gneg cells (T-cells, NK-cells, B-cells). F4/80neg, Ly6Gpos were confirmed to be neutrophils by additional gating for Ly-6C and CD11b expression. The F4/80pos, Ly6Gneg cells were plotted on a light scatter plot to identify eosinophils (SSC-Ahigh) and monocytes/macrophages (SSC-Alow). Finally, F4/80neg Ly6Gneg cells were represented on a CD19 versus CD3 plot to identify T-cells (CD3pos CD19neg) and B-cells (CD3neg CD19pos). Absolute cell counts were calculated by multiplying the total number of immune cells in 100μl of blood (after RBC depletion) with the percentage of each cell type determined by flow cytometry.

**CD31 Immunostaining**

CD31 stainings on 14 µm brain tissue cryosections were performed using a rat anti-mouse CD31 antibody (BD Biosciences), overnight, followed by donkey anti-rat antibody Alexa Fluor 555 labelled antibody (Abcam).

**Analysis of CD31 sections**

Images of the tumor stained for CD31 expression were acquired using a Leica DM6000 fluorescence microscope. Tumor vascularization analysis was carried out using the open-source ImageJ software. Briefly, for each section the tumor and the vessel area was identified and measured by applying a threshold on the corresponding channel and obtaining a binary image representative of its distribution. Relative tumor vascularization was quantified by normalizing the vascular area to the tumor area for each section.

**Bioluminescent monitoring and survival analysis (supplemental experiment)**

Bioluminescence imaging was performed after intraperitoneal injection of 150µl d–Luciferin (25mg/L, Gold Biotechnology) using the Xenogen IVIS 200 Imaging System (PerkinElmer). Prior to imaging mice are sedated with isofluorane and hair removed using scalpel. Clinical signs upon to mice were euthanized are 20% weight loss, dehydration, hunched posture and rough hair coat.

**SUPPLEMENTARY MOVIE LEGENDS**

Supplementary Movie 1. Intravital imaging video showing GL261-H2B Dendra2 cells migrating (red lines) and undergoing mitosis (white arrows) in a control animal. Left: tumor cells; middle: tumor cells + cell tracks; right: cell tracks. Scale bar represents 50 µm.

Supplementary Movie S. Intravital imaging video showing GL261-H2B Dendra2 cells migrating (red lines) and undergoing mitosis (white arrows) in a biopsied animal. Left: tumor cells; middle: tumor cells + cell tracks; right: cell tracks. Scale bar represents 50 µm.

**SUPPLEMENTARY FIGURES AND FIGURE LEGENDS**

**
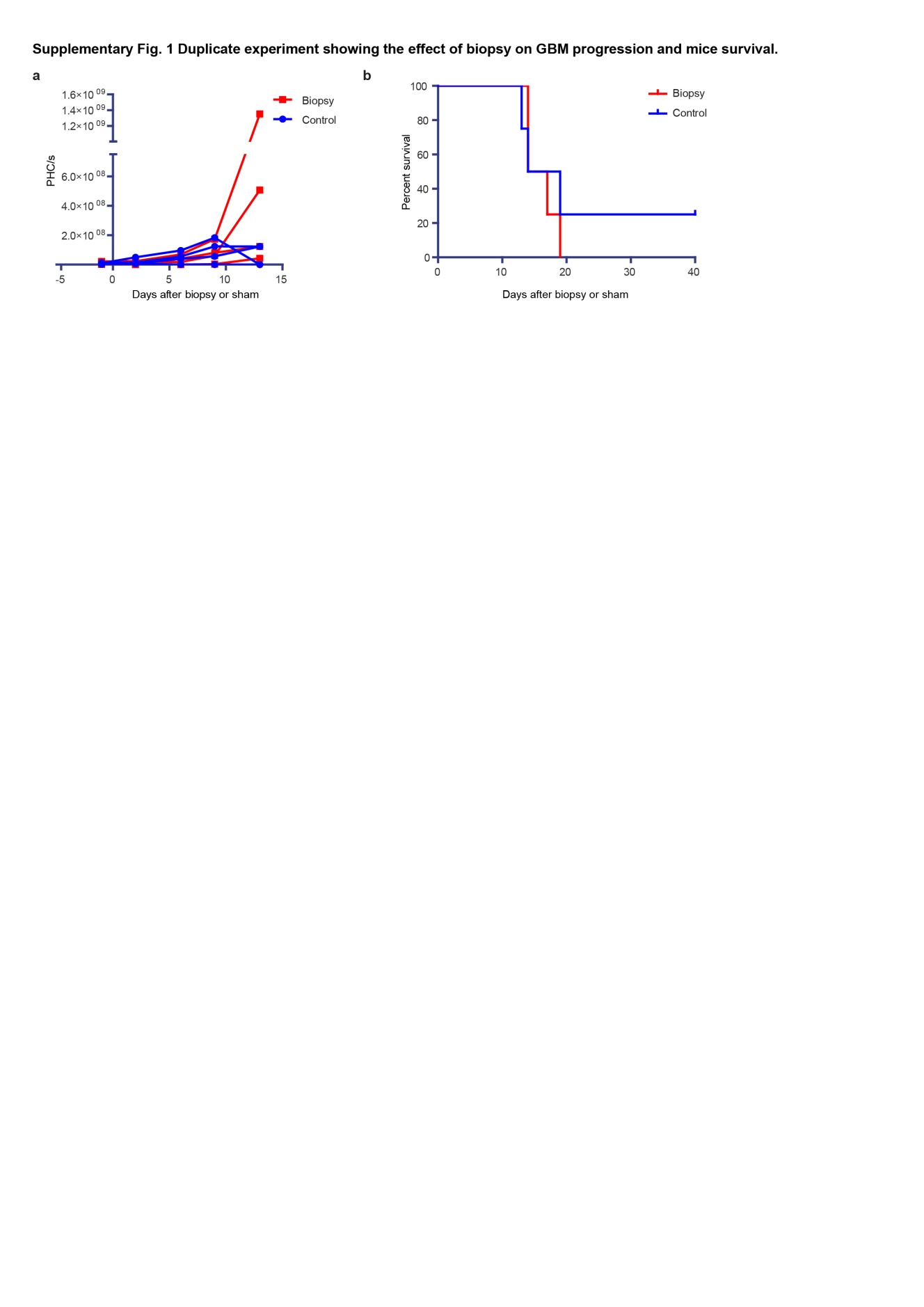
**

**Supplementary Fig. 1 Duplicate experiment showing the effect of biopsy on GBM progression and mice survival.** (**a**) Kinetics of glioma growth per indivudual mice. Each dot represents the photon counts per second (PHC/s) values from inidvidual mice, values from the same mouse are connected with a line (n=4). (**b**) Kaplan-Meier survival curves of mice that received a biopsy-like injury or control mice.

**
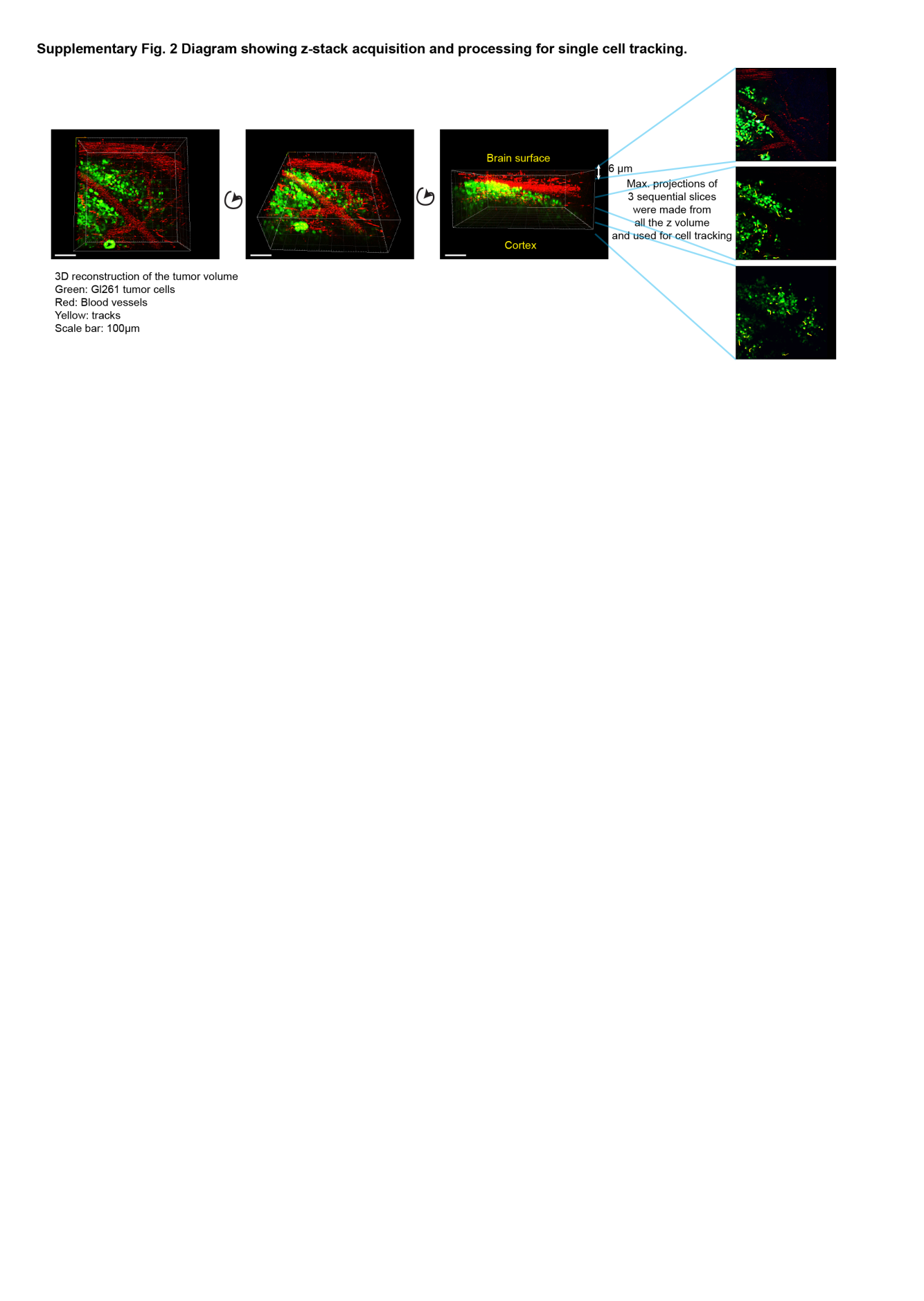
**

**Supplementary Fig. 2. Diagram showing z-stack acquisition and processing for single cell tracking.** For each position, images of the complete z stack of the tumor were acquired to a depth of 300 μm, with a step size of 3 µm. Throughout the complete z stack maximum projections of 3 consequent z planes were made for the analysis. At the beginning of each movie, a random cell was selected and up to 50/70 cells (for GBM and PyMT respectively) per imaging field of each z-projection were tracked manually.


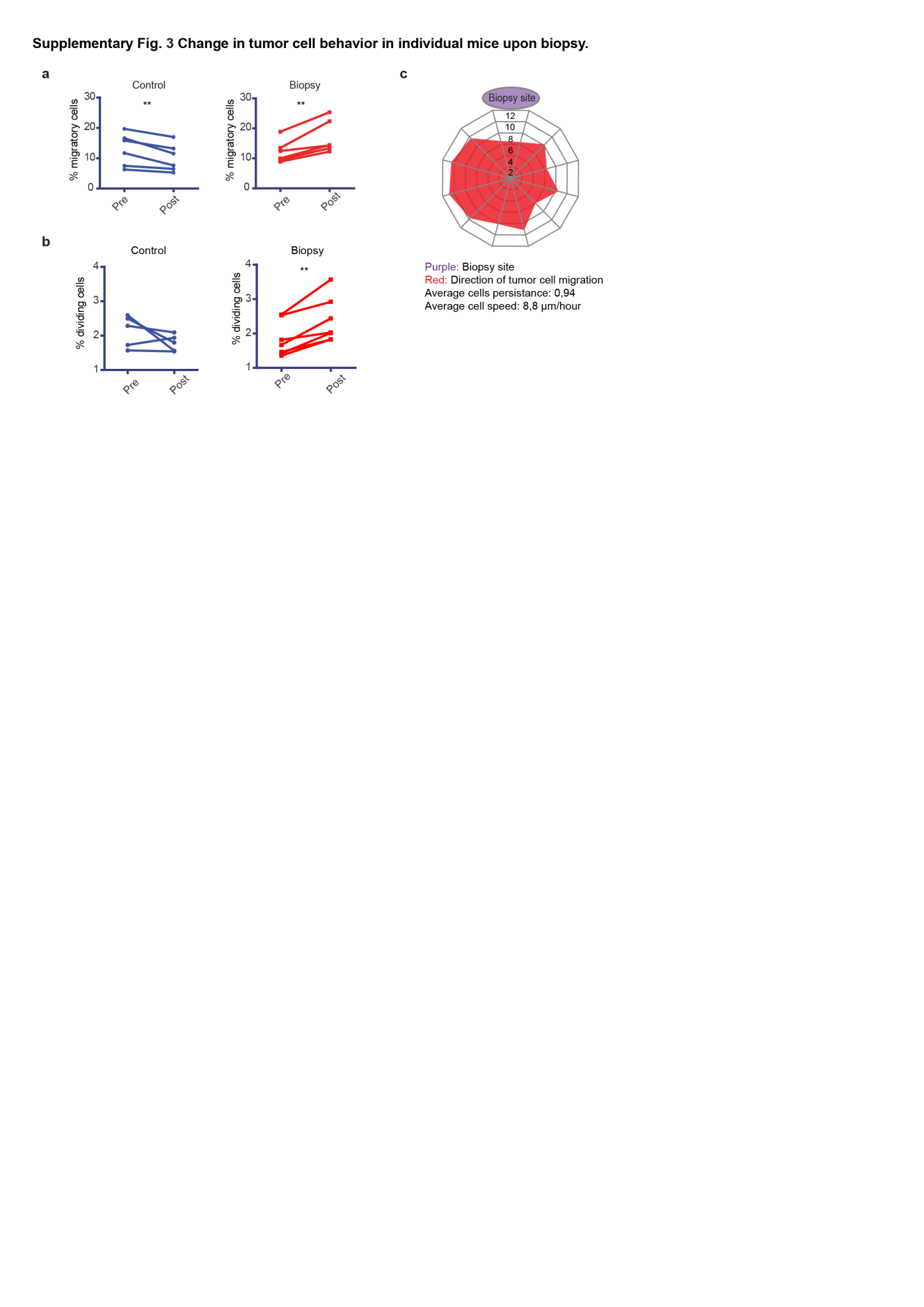


**Supplementary Fig. 3. Change in tumor cell behavior in individual mice upon biopsy.** (**a**) Percentage of migratory cells (velocity >4μm/hour) per individual mouse Pre and Post intervention. Values of the same animal are connected with a line. *n* = 6 mice. (**b**) Percentage of dividing cells per individual mouse Pre and Post intervention. Values of the same animal are connected with a line. *n* >= 5 mice. **P<0.01 versus control, Student’s paired *t* test). (**c**) Wind-rose plot representing the percentage of tumor cells migrating in the corresponding angular bin. Shown are mean values from biopsied positions from 3 different animals.

**
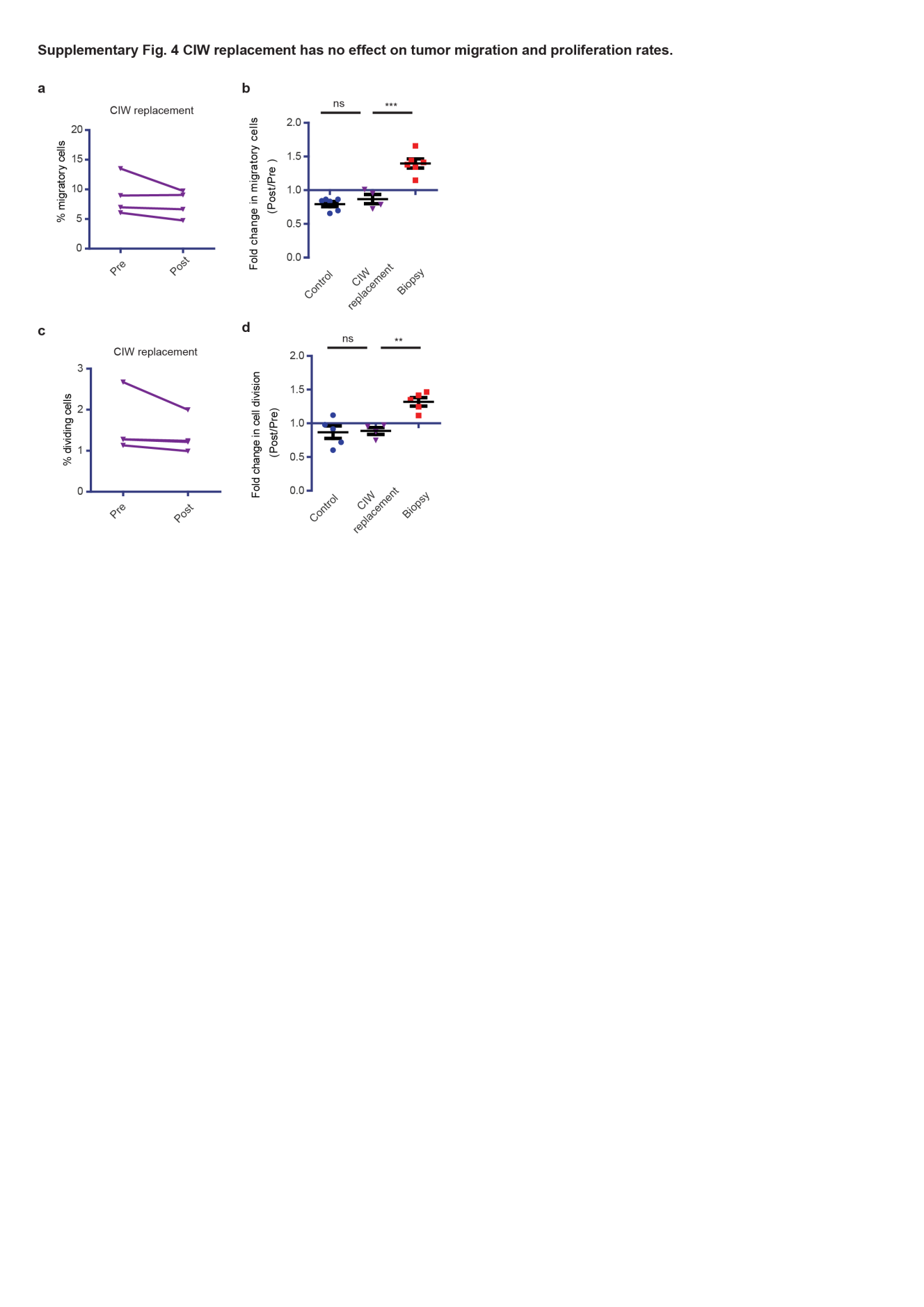
**

**Supplementary Fig. 4. CIW replacement has no effect on tumor migration and proliferation rates.** (**a**) Percentage of migratory cells per individual mouse Pre and Post intervention. Values of the same animal are connected with a line. *n* = 4 mice. (**b**) The increase in the number of migratory cells for the indicated conditions. Every symbol represents the mean of an individual mouse, and *n* >= 4 mice. (**c**) Percentage of dividing cells per individual mouse Pre and Post intervention. Values of the same animal are connected with a line. (**d**) The increase in the number of migratory cells for the indicated conditions. Every symbol represents the mean of an individual mouse. (*n* >= 4 mice,**P<0.01,***P<0.001 one-way ANOVA with Newman-Keuls’s post hoc test).

**
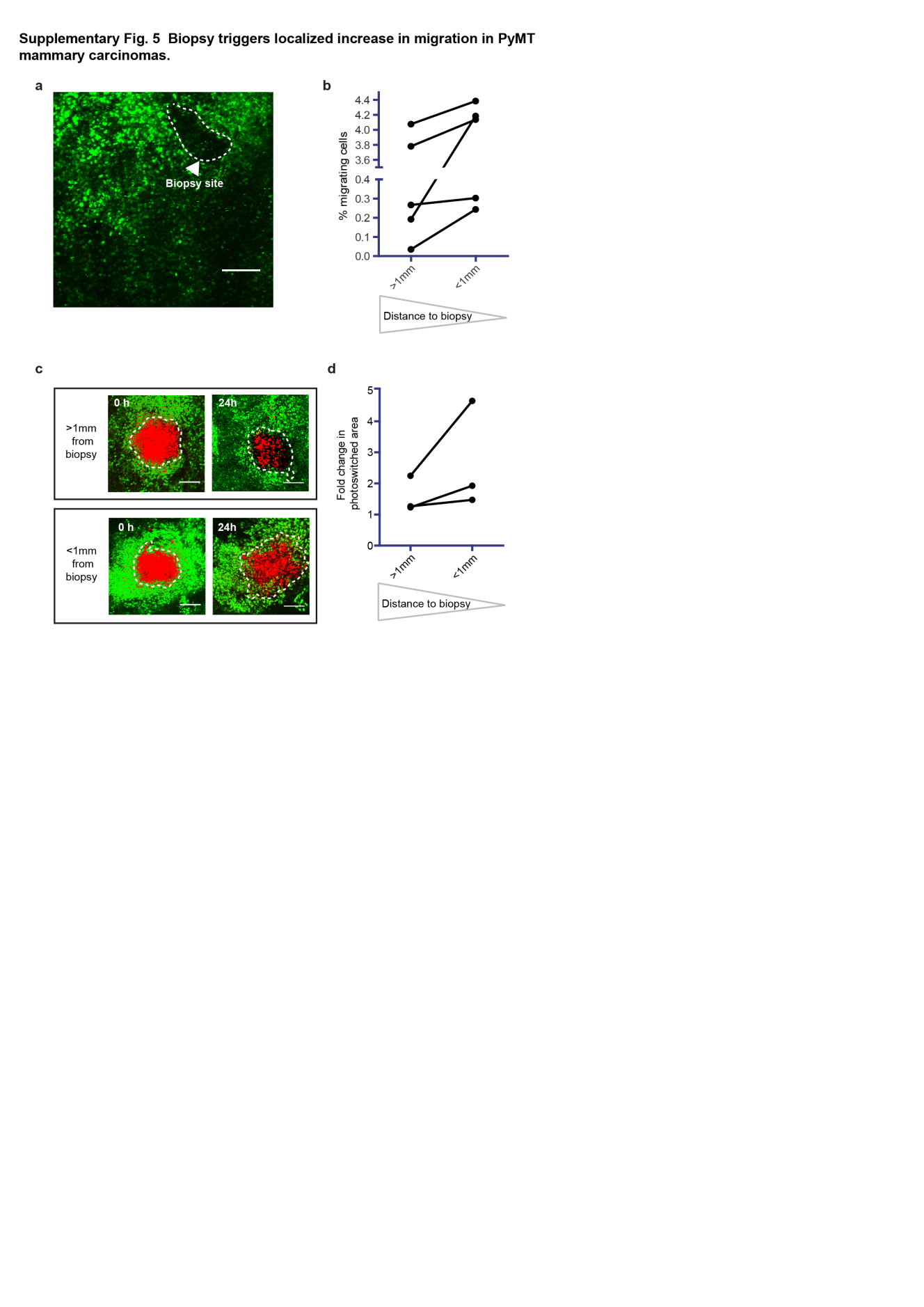
**

**Supplementary Fig. 5. Biopsy triggers localized increase in migration in PyMT mammary carcinomas.** (**a**) Maximum projection of a z stack showing the site of biopsy in a PyMT mammary carcinoma tumor. Scale bar represents 100 µm. (**b**) Percentage of migrating (>6µm/h) cells per individual mouse far (>1mm) and close (<1mm) from the biopsy site. Values of the same animal are connected with a line. *n* = 5 mice. (**c**) Representative Dendra2 images of tumor cell migration and infiltration, far (>1mm) and close (<1mm) from the biopsy site. The white dotted line represents the migration and infiltration area. Scale bar represents 100 µm. (**d**) The increased photoconverted area plotted far (>1mm) and close (<1mm) from the biopsy site. Values of the same animal are connected with a line. *n* = 3 mice.


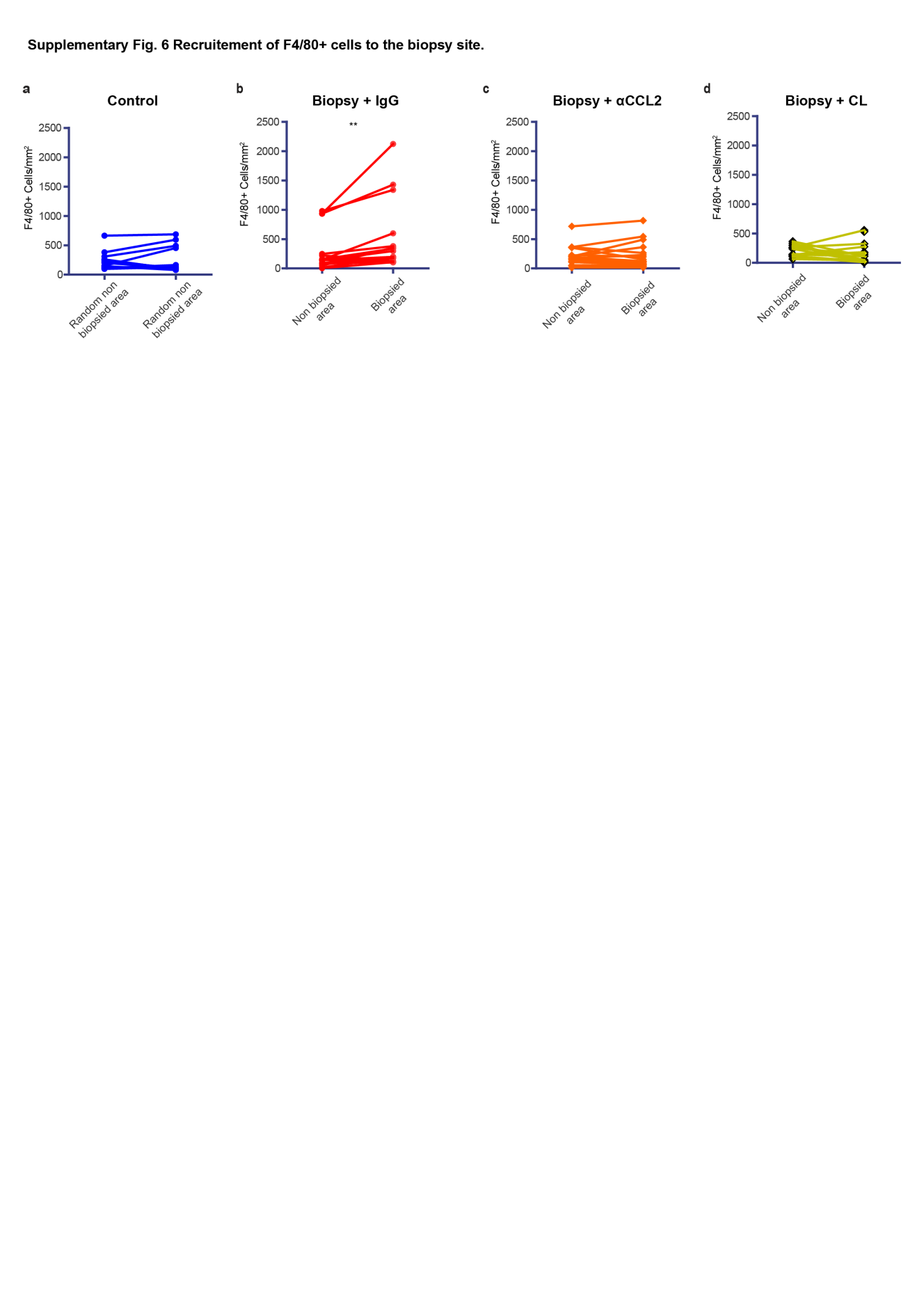


**Supplementary Fig. 6. Recruitment of F4/80+ cells to the biopsy site.** Raw numbers of F4/80+ cells/mm^2^ at the biopsied and non biopsied areas in control (**a**); biopsy + IgG antibody (**b**), biopsy + αCCL-2 antibody (**c**); and biopsy + clodronate liposomes (**d**) animals. Values from the same tumor slice are connected with a line. *n* >= 3 mice. **P<0.01 versus control, Student’s paired *t* test).

**
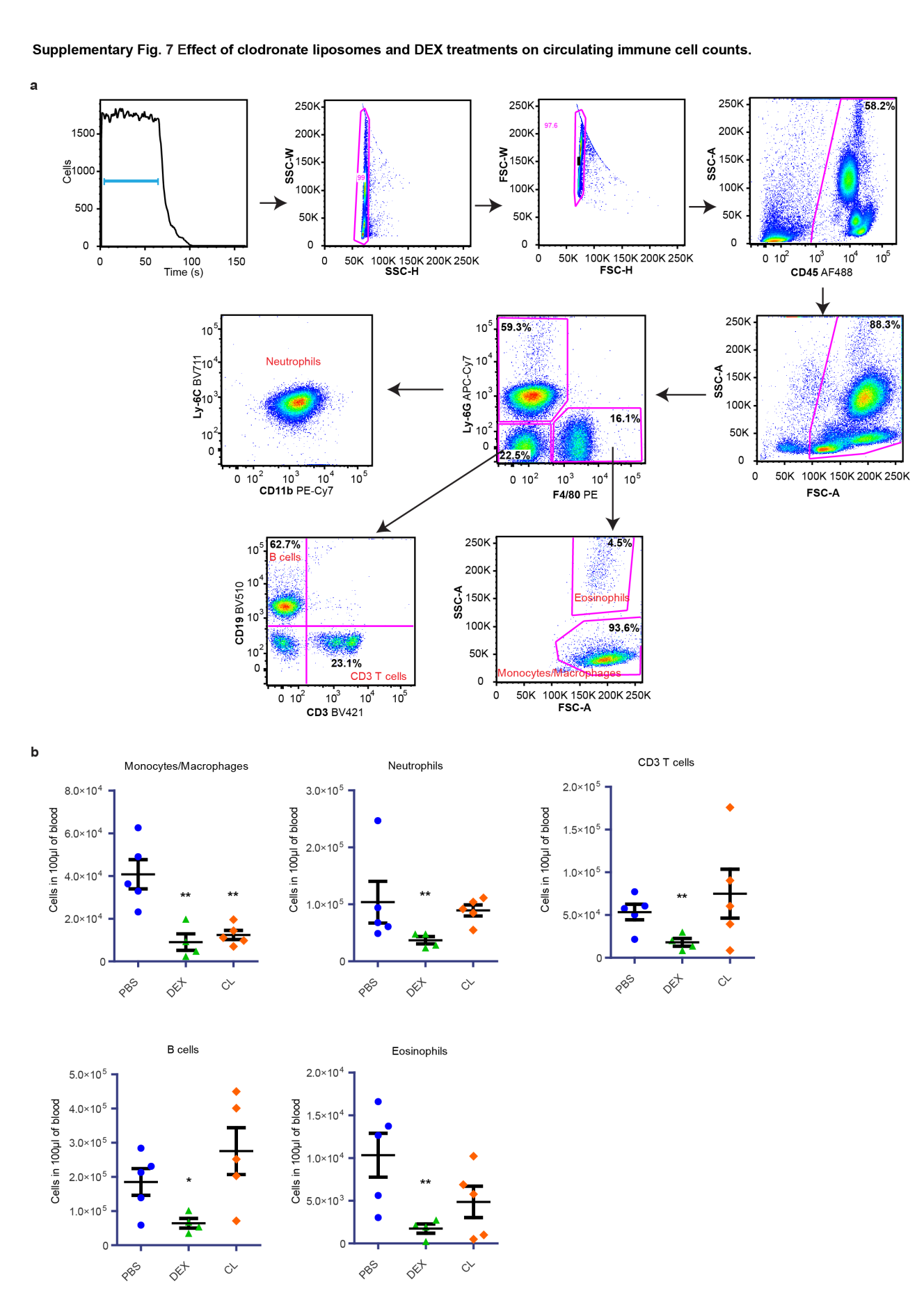
**

**Supplementary Fig. 7. Effect of clodronate liposomes and DEX treatments on circulating immune cell counts**. (**a**) Flow cytometry gating strategy for different cell populations in blood. Events acquired over stable flow were selected, followed by a gate excluding doublets. Immune cells were gated based on CD45 expression. A light scatter gate was constructed to exclude debris and non-viable cells from the analysis. The remaining events were represented in a F4/80 versus Ly-6G plot to identify neutrophils (F4/80^neg^, Ly6G^pos^), F4/80^pos^, Ly6G^neg^  (monocytes/macrophages and eosinophils) and F4/80^neg^ Ly6G^neg^ cells (T-cells, NK-cells, B-cells). F4/80^neg^, Ly6G^pos^ were confirmed to be neutrophils by additional gating for Ly-6C and CD11b expression. The F4/80^pos^, Ly6G^neg^ cells were plotted on a light scatter plot to identify eosinophils (SSC-A^high^) and monocytes/macrophages (SSC-A^low^). Finally, F4/80^neg^ Ly6G^neg^ cells were represented on a CD19 versus CD3 plot to identify T-cells (CD3^pos^ CD19^neg)^ and B-cells (CD3^neg^ CD19^pos^). (**b**) Absolute numbers of monocytes/macrophages, neutrophils, T-cells, B-cells, and eosinophils in control (blue), DEX treated (green) and clodronated liposomes (CL) treated (orange) mice. Every symbol represents an individual mouse. (*n* >= 5 mice, *P<0.05, **P<0.01 versus control, Student’s *t* test).

**
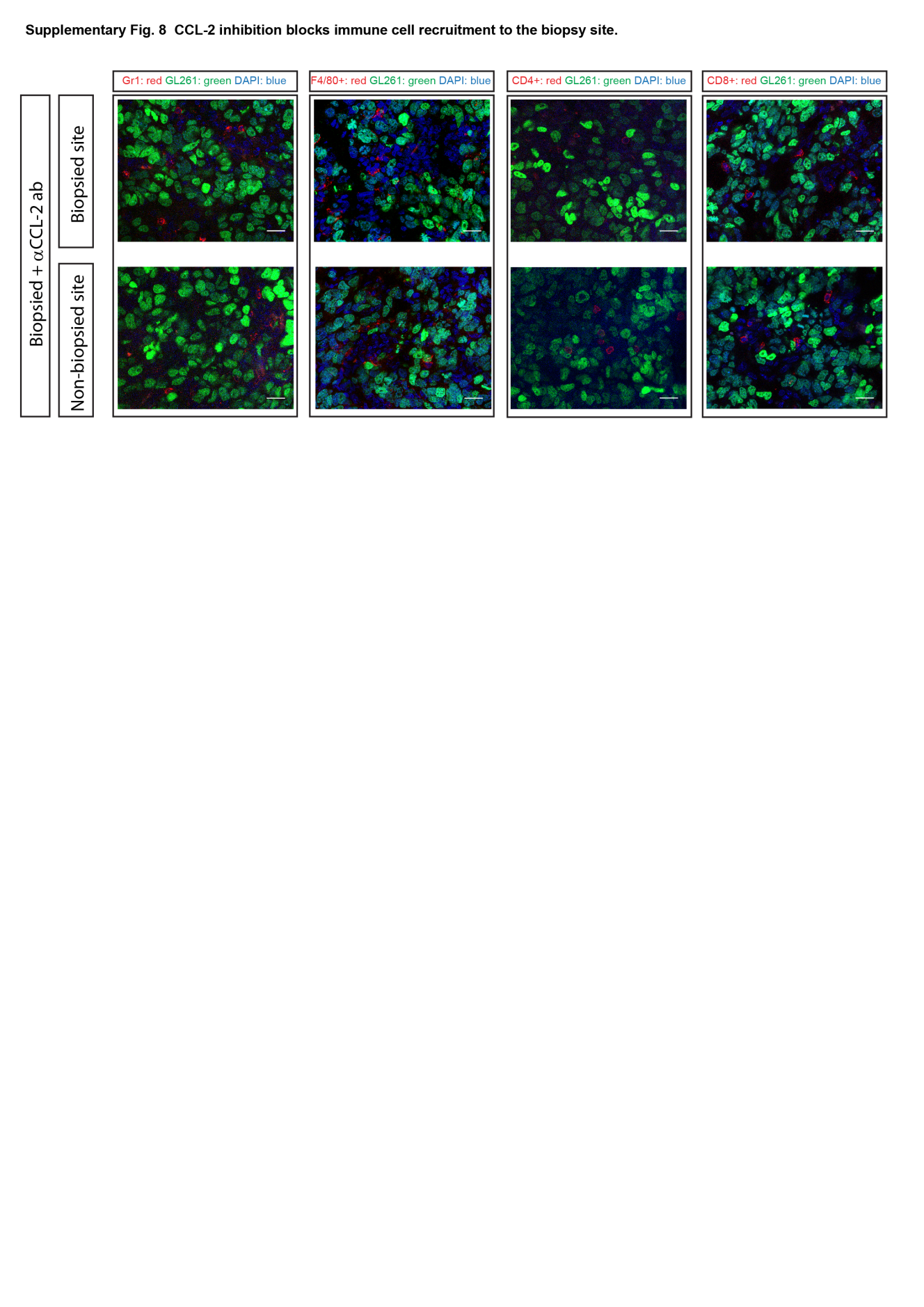
**

**Supplementary Fig. 8. CCL-2 inhibition blocks immune cell recruitment to the biopsy site.** Representative staining showing Gr-1 (Ly-6G)+ neutrophils; F4/80+ macrophages/microglia; CD4+ lymphocytes; CD8+ lymphocytes at biopsied and non biopsied sites of mice that were injected with a CCL-2 blocking antibody (upper). Shown are immune cell stainings in red, H2B-Dendra2 GL261 expression in green and DAPI staining in blue. Scale bar represents 20 μm.


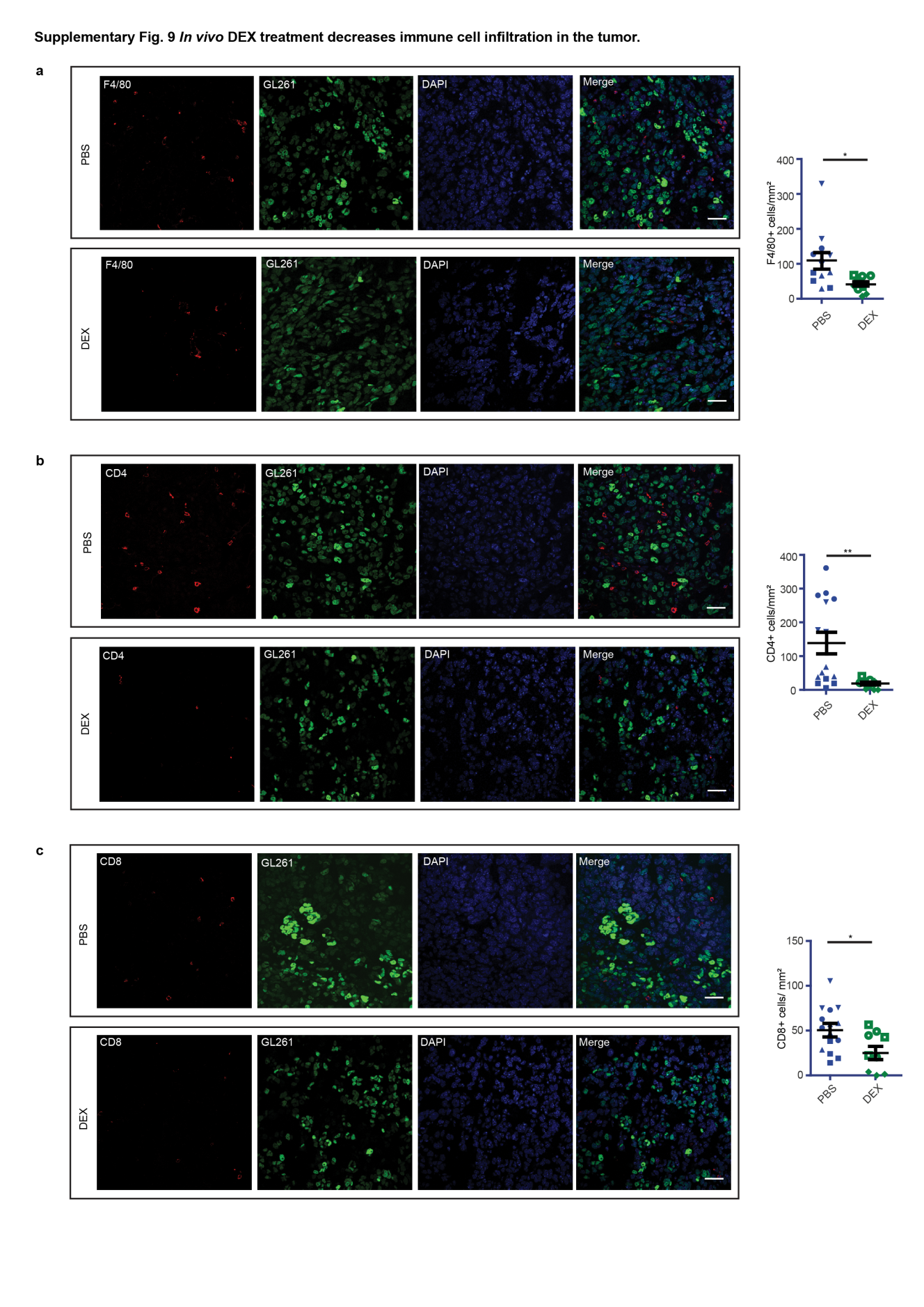


**Supplementary Fig. 9. *In vivo* DEX treatment decreases immune cell infiltration in the tumor.** (**a**-**c**) F4/80+ macrophages/microglia (a); CD4+ lymphocytes (b**)**; CD8+ lymphocytes (c) staining of the tumor mass upon DEX treatment (bottom) as compared to PBS treated mice (top) assessed by confocal microscopy. Shown are immune cell stainings in red, H2B-Dendra2 GL261 expression in green and DAPI staining in blue. Scale bar represents 40 μm. Graphs show immune cell counts per tumor area in mice treated for 5 days with PBS or DEX. Each dot represents values from one brain slice, and the different symbols represent different animals. The data is shown as mean ± S.E.M. (*n* >= 3 mice, *P<0.05 versus control, Student’s *t* test).


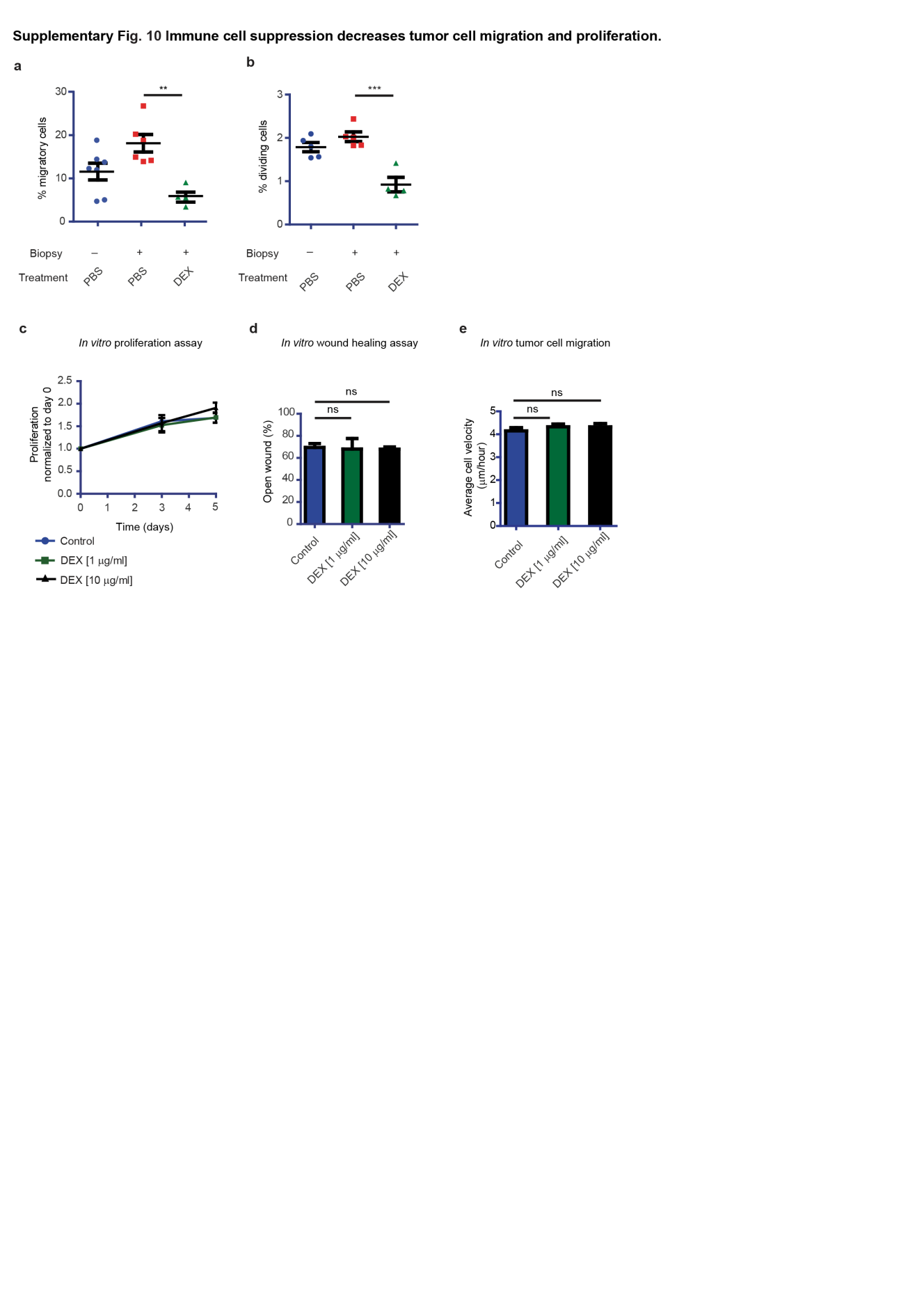


**Supplementary Fig. 10. Immune cell suppression decreases tumor cell migration and proliferation**. (**a**,**b**) Graphs showing percentage of migratory (a) and dividing (b) tumor cells in the indicated experimental groups. Each dot represents values from individual mice. (*n* >= 4 mice, **P<0.01, ***<0.001, one-way ANOVA with Newman-Keuls’s post hoc test). (**c**) *In vitro* cell proliferation of GL261 cells cultured with PBS or DEX. Data is based on triplicates. (**d**) *In vitro* GL261 invasion measured by scratch assay of cells cultured with PBS or DEX. Data is based on triplicates. (**e**) Graph showing the average velocity of individual GL261 cells tracked *in vitro*, cultured with PBS or DEX. Data is based on triplicates. (One-way ANOVA with Newman-Keuls’s post hoc test).

**
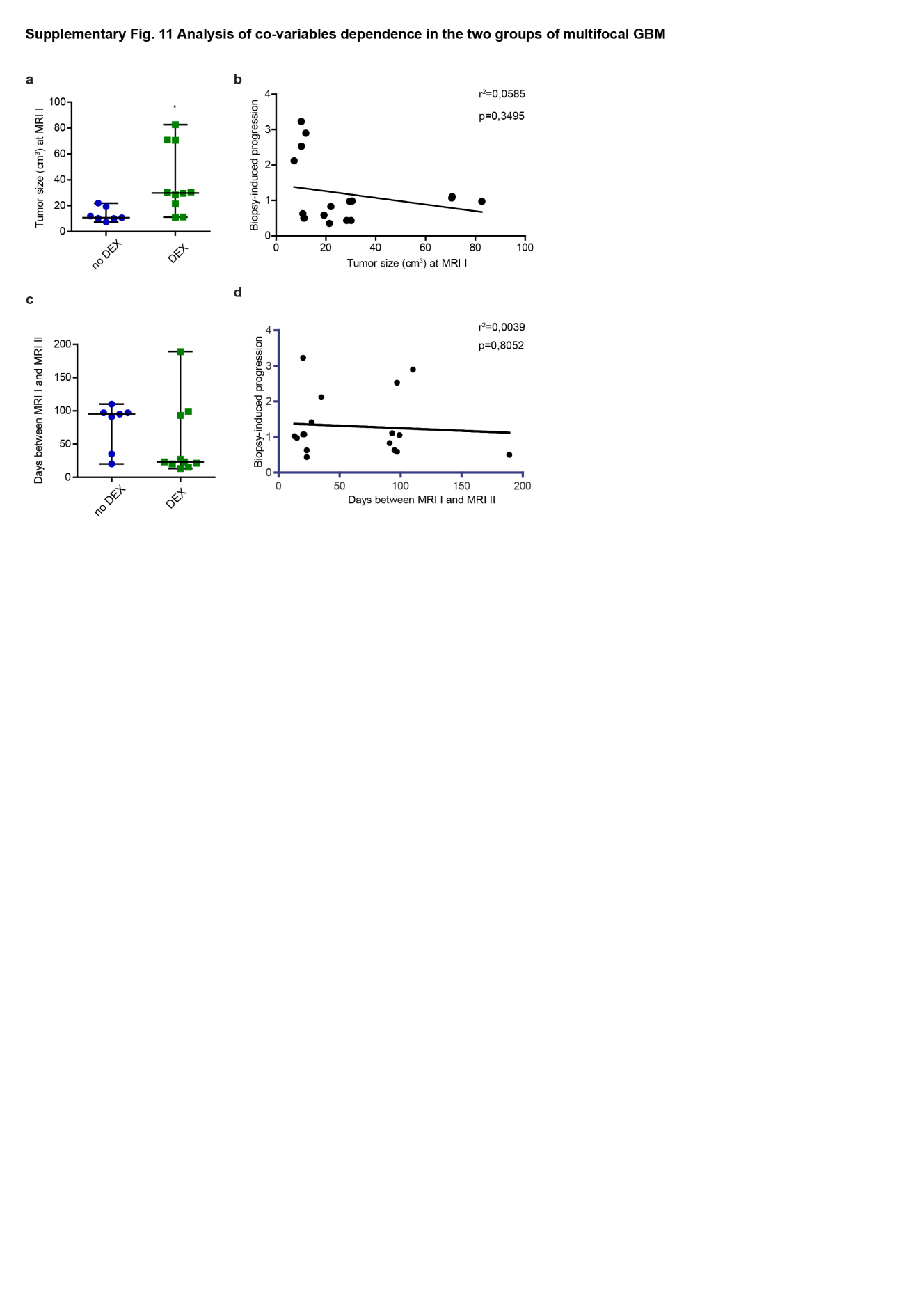
**

**Supplementary Fig. 11.** **Analysis of co-variables dependence in the two groups of multifocal GBM patients.** (**a**) Initial tumor volume from patients that do not receive DEX treatments before biopsy (blue) and patients that receive DEX before biopsy (green) before biopsy (MRI I). (**b**) Correlation between initial tumor size (before biopsy) and biopsy-induced tumor cell progression, normalized to its non-biopsied counterpart. (**c**) Days between MR measurements in patients that did not receive DEX treatments before biopsy (blue) and patients that receive DEX before biopsy (green).(**d**) Correlation between days between MR measurements and biopsy-induced tumor cell progression, normalized to its non-biopsied counterpart. (n=17, P value determined with Pearson correlation analysis).


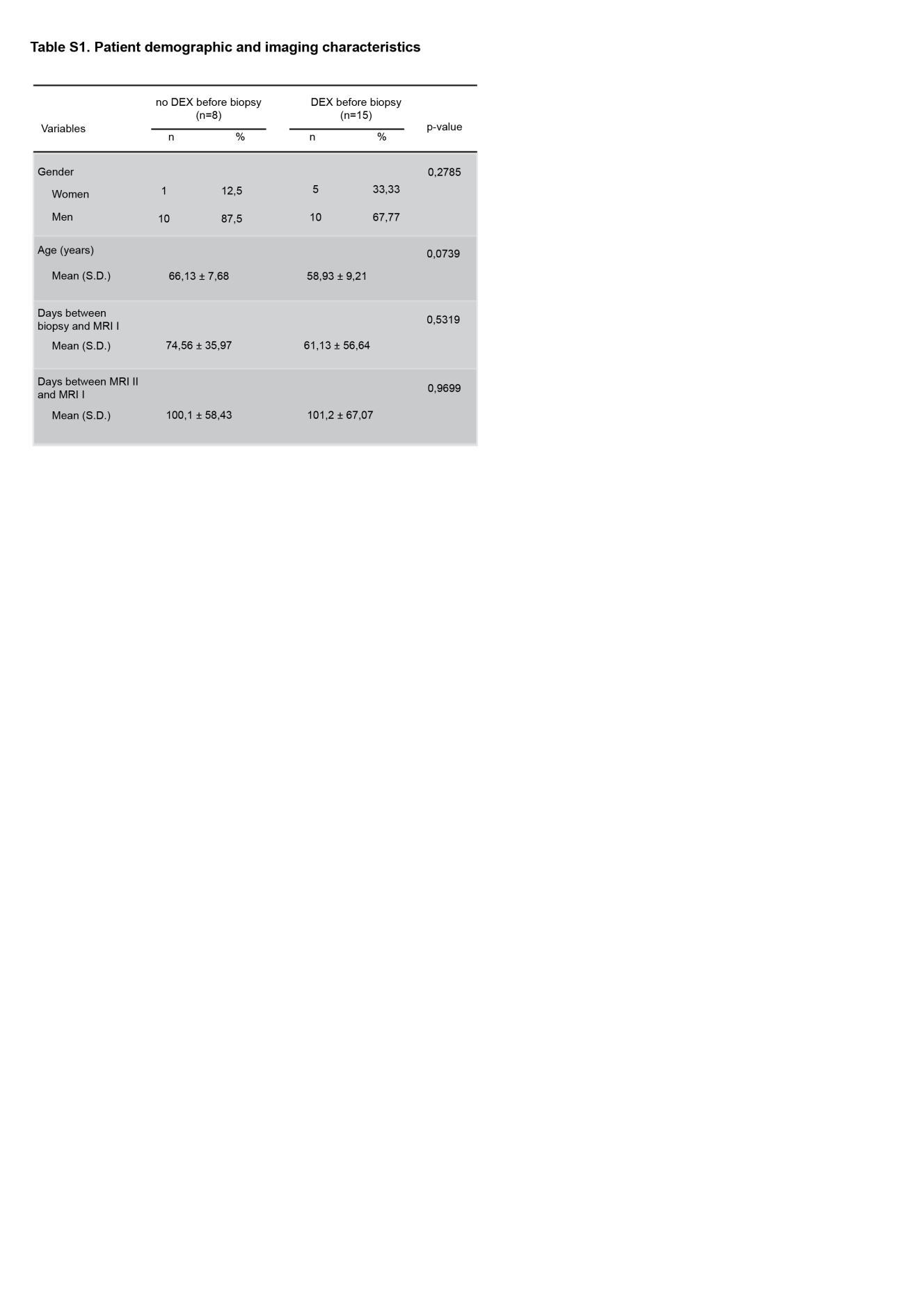


**Supplementary Table 1. Patient demographic and imaging characteristics.**
